# The airway smooth muscle sodium/calcium exchanger NCLX is critical for airway remodeling and hyperresponsiveness in asthma

**DOI:** 10.1016/j.jbc.2022.102259

**Published:** 2022-07-14

**Authors:** Martin T. Johnson, J. Cory Benson, Trayambak Pathak, Ping Xin, Abagail S. McKernan, Scott M. Emrich, Ryan E. Yoast, Vonn Walter, Adam C. Straub, Mohamed Trebak

**Affiliations:** 1 Department of Cellular and Molecular Physiology, The Pennsylvania State University College of Medicine, Hershey, Pennsylvania, USA; 2 Department of Pharmacology and Chemical Biology, University of Pittsburgh School of Medicine, Pittsburgh, Pennsylvania, USA; 3 Vascular Medicine Institute, University of Pittsburgh School of Medicine, Pittsburgh, Pennsylvania, USA; 4 Department of Public Health Sciences, The Pennsylvania State University College of Medicine, Hershey, Pennsylvania, USA

**Keywords:** calcium signaling, mitochondrial calcium, NCLX, SOCE, metabolism, CaMKII, asthma, airway remodeling, airway fibrosis, airway hyperresponsiveness, 7-AAD, 7-aminoactinomycin D, AGC, automatic gain control, AHR, airway hyperresponsiveness, AR, airway remodeling, ASM, airway smooth muscle, BAL, bronchoalveolar lavage, CaMKII, Ca^2+^/calmodulin-dependent kinase II, cDNA, complementary DNA, ER, endoplasmic reticulum, FCCP, carbonyl cyanide-4-(trifluoromethoxy)phenylhydrazone, GSEA, gene set enrichment analysis, HASMC, human airway smooth muscle cell, HBSS, Hepes-buffered salt solution, HDM, house dust mite, IgE, immunoglobin E, IL-6, interleukin 6, IP_3_, inositol-1,4,5-trisphosphate, KD, knockdown, LC3B, light chain 3B, MASMC, mouse airway smooth muscle cell, MCU, mitochondrial Ca^2+^ uniporter, MeCh, methacholine, mPTP, mitochondrial permeability transition pore, mtDNA, mitochondrial DNA, NCLX, Na^+^/Ca^2+^ exchanger, NFAT, nuclear factor of activated T cell, PM, plasma membrane, qPCR, quantitative PCR, ROI, region of interest, α-SMA, α-smooth muscle actin, SOCE, store-operated Ca^2+^ entry, STIM, stromal interaction molecule, TBST, Tris-buffered saline with Tween-20, TCA, tricarboxylic acid, TMRE, tetramethylrhodamine, ethyl ester, VSMC, vascular smooth muscle cell

## Abstract

The structural changes of airway smooth muscle (ASM) that characterize airway remodeling (AR) are crucial to the pathogenesis of asthma. During AR, ASM cells dedifferentiate from a quiescent to a proliferative, migratory, and secretory phenotype. Calcium (Ca^2+^) is a ubiquitous second messenger that regulates many cellular processes, including proliferation, migration, contraction, and metabolism. Furthermore, mitochondria have emerged as major Ca^2+^ signaling organelles that buffer Ca^2+^ through uptake by the mitochondrial Ca^2+^ uniporter and extrude it through the Na^+^/Ca^2+^ exchanger (NCLX/Slc8b1). Here, we show using mitochondrial Ca^2+^–sensitive dyes that NCLX only partially contributes to mitochondrial Ca^2+^ extrusion in ASM cells. Yet, NCLX is necessary for ASM cell proliferation and migration. Through cellular imaging, RNA-Seq, and biochemical assays, we demonstrate that NCLX regulates these processes by preventing mitochondrial Ca^2+^ overload and supporting store-operated Ca^2+^ entry, activation of Ca^2+^/calmodulin-dependent kinase II, and transcriptional and metabolic reprogramming. Using small animal respiratory mechanic measurements and immunohistochemistry, we show that smooth muscle–specific NCLX KO mice are protected against AR, fibrosis, and hyperresponsiveness in an experimental model of asthma. Our findings support NCLX as a potential therapeutic target in the treatment of asthma.

Asthma is a chronic inflammatory disease of the lungs that affects approximately 339 million people globally and is a significant burden on the health care system costing the United States approximately 81.9 billion dollars a year (1). Symptoms of asthma include episodic shortness of breath, wheezing, chest tightness, and cough. Current treatment modalities aim at relieving inflammation and dilating the airways. However, there are still many patients with these conventional therapies who continue to display uncontrolled asthma symptoms (2). Furthermore, pulmonary function continues to decline in many asthmatic patients, highlighting the need for additional studies to further understand the molecular mechanisms of asthma and identify novel drug targets.

It is well established that inflammation is involved in the pathogenesis of asthma. However, evidence is emerging that the pathogenesis of asthma is also because of structural changes in the airways, termed airway remodeling (AR). Both mild and severe asthmatic patients have AR, and the severity of AR correlates with the severity of symptoms (3, 4). AR includes goblet cell hyperplasia and metaplasia, fibrosis, thickening of the lamina reticularis, angiogenesis, and hyperplasia and hypertrophy of airway smooth muscle (ASM) (5). ASM cells are major effectors of AR because of their ability to dedifferentiate from a contractile/quiescent phenotype into a highly proliferative, migratory, and secretory phenotype. This “phenotypic switch” from quiescent to synthetic smooth muscle ultimately causes worse disease outcomes (6, 7).

Accompanying this phenotypic switch is a major molecular reprogramming in the calcium (Ca^2+^) signaling machinery (8). Ca^2+^ is a ubiquitous secondary messenger that regulates smooth muscle contraction, metabolism, and activation of proliferative and migratory transcriptional programs (8, 9). In synthetic smooth muscle cells, Ca^2+^ signaling pathways that favor proliferation and migration are upregulated. Our group and others have previously shown that the store-operated Ca^2+^ entry (SOCE) pathway is upregulated during smooth muscle remodeling and is required for the progression of both asthma and cardiovascular diseases (10, 11, 12, 13, 14, 15, 16). SOCE is encoded by stromal interaction molecule (STIM) and Orai proteins and activated upon depletion of endoplasmic reticulum (ER) Ca^2+^ stores by physiological agonists. Upon store depletion, the Ca^2+^-sensing STIM proteins in the ER aggregate and move to ER–plasma membrane (PM) junctions, and physically trap PM Orai Ca^2+^ channels, triggering Ca^2+^ influx from the extracellular space. This Ca^2+^ influx route activates fibroproliferative signaling pathways that drive smooth muscle remodeling (16, 17, 18). We recently showed that STIM1 upregulation in ASM of asthmatic mice drives transcriptional and metabolic reprogramming and is a central determinant for ASM remodeling and hyperresponsiveness in asthma (16).

Although classically described as the powerhouse of the cell, mitochondria have also emerged as significant Ca^2+^ signaling organelles (19, 20). By positioning themselves close to ER Ca^2+^ release channels like the inositol-1,4,5-triphosphate receptors and Orai channels at the PM, mitochondria can buffer incoming Ca^2+^ into the mitochondrial matrix through the mitochondrial Ca^2+^ uniporter (MCU) channel complex. This buffering by mitochondria helps shape cytosolic Ca^2+^ signals that target Ca^2+^-effector proteins (21, 22). Furthermore, increased Ca^2+^ in the mitochondrial matrix stimulates Ca^2+^-sensitive enzymes of the citric acid (tricarboxylic acid [TCA]) cycle and complexes in the electron transport chain to enhance metabolism (20, 23, 24, 25). Specifically, mitochondrial mass and respiration are increased in ASM of asthmatic patients (16, 26, 27). The mitochondrial Na^+^/Ca^2+^ exchanger (NCLX)–mediated mitochondrial Ca^2+^ extrusion is critical for preventing mitochondrial Ca^2+^ overload and subsequent opening of mitochondrial permeability transition pore (mPTP). Indeed, inducible cardiac-specific NCLX KO mice suffer from sudden cardiac death because of mitochondrial Ca^2+^ overload (28). Recent studies have demonstrated that NCLX function is critical for cellular proliferation and migration of colorectal cancer cells, B lymphocytes, and astrocytes (29, 30, 31). We previously showed that NCLX downregulation in vascular smooth muscle cells (VSMCs) causes mitochondrial Ca^2+^ overload, enhanced production of mitochondrial hydrogen peroxide, and subsequent inhibition of SOCE through oxidation of cysteine 195 on Orai1 channels (32). However, the role of NCLX in ASM remodeling during asthma is unknown.

Here, we show that although NCLX has only a moderate contribution to mitochondrial Ca^2+^ extrusion, its function supports optimal SOCE activity in ASM cells. We show that NCLX is critical for preventing ASM apoptosis and for driving ASM cell proliferation and migration through stimulation of proremodeling transcriptional and metabolic programs and activation of Ca^2+^/calmodulin-dependent kinase II (CaMKII). We reveal that ASM NCLX is necessary for AR and airway hyperresponsiveness (AHR) in asthmatic mice. Our results show that ASM NCLX is critical for AR and AHR in asthma and suggest NCLX as a potential target in the treatment of asthma.

## Results

### NCLX is required for optimal SOCE function in human ASM cells

**Figure 1:**
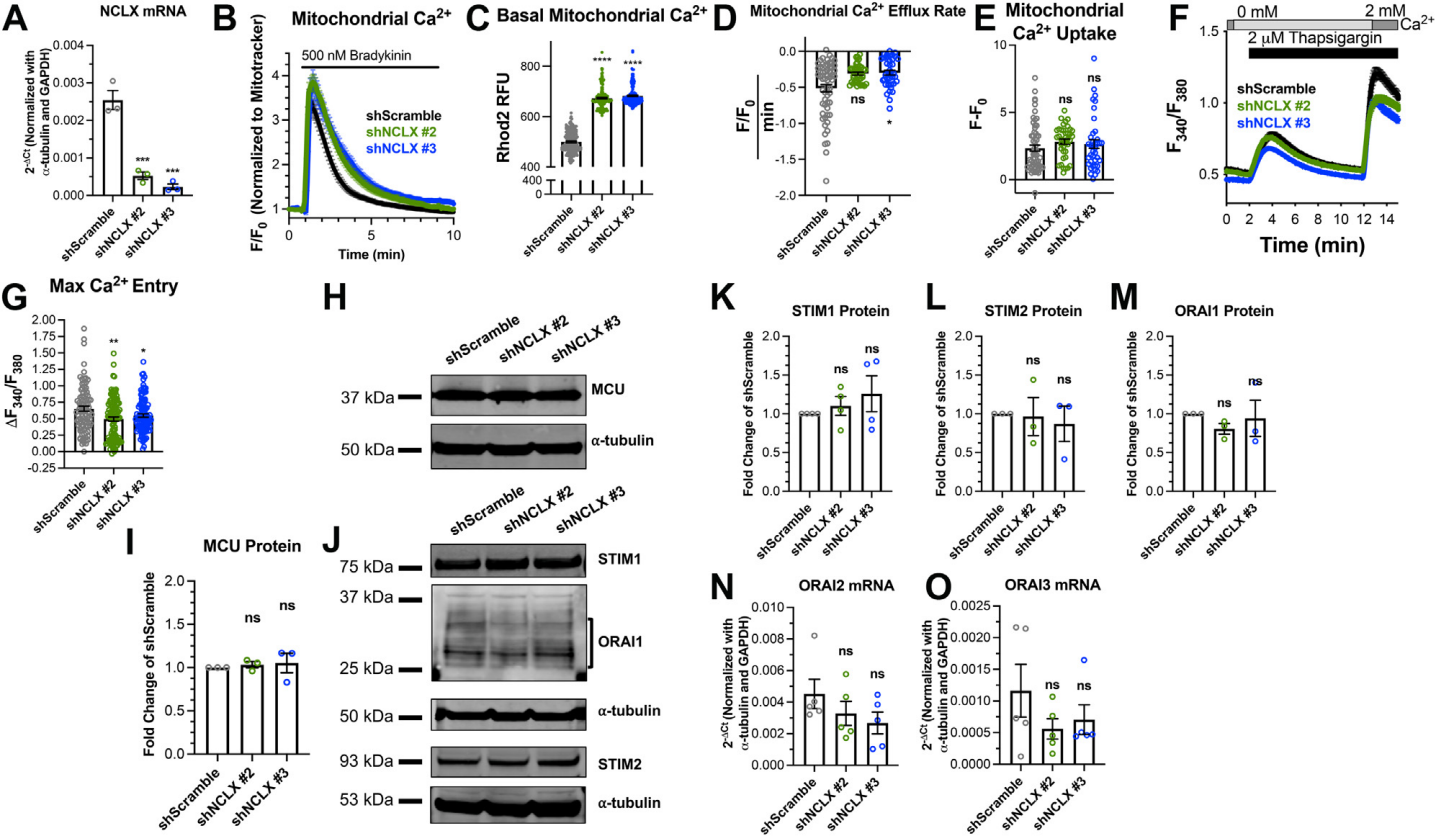
**NCLX regulates SOCE in HASMCs.***A*, quantification of NCLX mRNA expression in shScramble (n = 3), shNCLX #2 (n = 3), and shNCLX #3 (n = 3) HASMCs using RT–qPCR (one-way ANOVA, *F*[2,6] = 59.18, *p* = 0.0001, with Dunnett’s post hoc test, *p* = 0.0003 for shNCLX #2 and *p* = 0.0001 for shNCLX #3). *B*, mitochondrial Ca^2+^ measurements in response to 500 nM bradykinin in the presence of 2 mM Ca^2+^ in shScramble (*black trace*) (n = 59), shNCLX #2 (*green trace*) (n = 39), and shNCLX #3 (*blue trace*) (n = 39) HASMCs. Mitochondrial Ca^2+^ is measured by dividing the fluorescence of Rhod-2 by MitoTracker Green. *C*, quantification of basal mitochondrial Ca^2+^ in the absence of agonist stimulation using Rhod-2/MitoTracker Green fluorescence (one-way ANOVA, *F*[2537] = 1151, *p* < 0.0001, with Dunnett’s post hoc test, *p* < 0.0001 for shNCLX #2 and *p* < 0.0001 for shNCLX #3). Quantification of (*D*) mitochondrial Ca^2+^ efflux rate (one-way ANOVA, Kruskal–Wallis statistic = 7.321, *p* = 0.0269, with Dunn’s post hoc test, *p* = 0.1174 for shNCLX #2 and *p* = 0.0244 for shNCLX #3) and (*E*) mitochondrial Ca^2+^ uptake (one-way ANOVA, Kruskal–Wallis statistic = 4.870, *p* = 0.0876, with Dunn’s post hoc test, *p* = 0.0578 for shNCLX #2 and *p* > 0.9999 for shNCLX #3) from *B*. *F*, cytosolic Ca^2+^ measurements using the standard Ca^2+^ off/Ca^2+^ on protocol with 2 μM thapsigargin in shScramble (*black trace*) (n = 95), shNCLX #2 (*green trace*) (n = 109), and shNCLX #3 (*blue trace*) (n = 103) HASMCs. *G*, quantification of maximal Ca^2+^ entry from *E* (one-way ANOVA, *F*[2304] = 6.096, *p* = 0.0025, with Dunnett’s post hoc test, *p* < 0.0013 for shNCLX #2 and *p* = 0.0446 for shNCLX #3). *H*, representative Western blots showing MCU protein expression in HASMCs transfected with shScramble and two NCLX shRNA (shNCLX #2 and shNCLX #3). (Note that the α-tubulin blot in *H* was reused for the α-tubulin blot for Figure 5*C*. The molecular weights for MCU and HIF1α were far enough to allow cutting and separate immunoblotting from the same blot). *I*, quantification of MCU protein levels in shScramble (n = 3), shNCLX #2 (n = 3), and shNCLX #3 (n = 3) HASMCs from *H* using densitometry normalized to α-tubulin (one-way ANOVA, *F*[2,6] = 0.1683, *p* = 0.8490, with Dunnett’s post hoc test, *p* = 0.9106 for shNCLX #2 and *p* = 0.7975 for shNCLX #3). *J*, representative Western blot showing STIM1, STIM2, and Orai1 protein expression in shScramble, shNCLX #2, and shNCLX #3 HASMCs. Quantification of (*K*) STIM1 (one-way ANOVA, *F*[2,9] = 0.7429, *p* = 0.5028, with Dunnett’s post hoc test, *p* = 0.8524 for shNCLX #2 and *p* = 0.4093 for shNCLX #3), (*L*) STIM2 (one-way ANOVA, *F*[2,6] = 0.1205, *p* = 0.8885, with Dunnett’s post hoc test, *p* = 0.9879 for shNCLX #2 and *p* = 0.8538 for shNCLX #3), and (*M*) Orai1 (one-way ANOVA, *F*[2,6] = 0.4989, *p* = 0.6304, with Dunnett’s post hoc test, *p* = 0.5532 for shNCLX #2 and *p* = 0.9385 for shNCLX #3) protein expression from *C* in shScramble (n = 3), shNCLX #2 (n = 3), and shNCLX #3 (n = 3) HASMCs using densitometry normalized to α-tubulin. Quantification of (*N*) Orai2 (one-way ANOVA, *F*[2,12] = 1.375, *p* = 0.2899, with Dunnett’s post hoc test, *p* = 0.4641 for shNCLX #2 and *p* = 0.2198 for shNCLX #3) and (*O*) Orai3 (one-way ANOVA, *F*[2,12] = 1.375, *p* = 0.2899, with Dunnett’s post hoc test, *p* = 0.4641 for shNCLX #2 and *p* = 0.2198 for shNCLX #3) mRNA expression in shScramble (n = 5), shNCLX #2 (n = 5), and shNCLX #3 (n = 5) HASMCs using RT–qPCR. ∗*p* < 0.05, *∗∗p* < 0.01, *∗∗∗p* < 0.001, and ns. HASMC, human airway smooth muscle cell; HIF1α, hypoxia-inducible factor 1α; MCU, mitochondrial Ca^2+^ uniporter; NCLX, Na^+^/Ca^2+^ exchanger; ns, not significant; qPCR, quantitative PCR; SOCE, store-operated Ca^2+^ entry.

We previously showed that abrogation of NCLX with siRNA inhibits SOCE and its biophysical manifestation, the Ca^2+^ release–activated Ca^2+^ current in primary VSMCs (32). Abrogation of mitochondrial Ca^2+^ extrusion causes mitochondrial Ca^2+^ overload and enhanced production of mitochondrial hydrogen peroxide, which in turn inhibit SOCE through oxidation of cysteine 195 on Orai1 channels (32). Here, we used human ASM cells (HASMCs) cultured from nonasthmatic donors (16, 33). Like VSMCs, when HASMCs are isolated and placed in cell culture with media containing growth factors from fetal bovine serum, they dedifferentiate from a contractile/quiescent to a synthetic phenotype, which remodels its cellular Ca^2+^ signaling machinery (6, 8, 12, 16, 34, 35). To determine the role of NCLX in regulating Ca^2+^ signaling and cellular function of HASMCs, we generated stable NCLX knockdown (KD) HASMCs using two different shRNAs (shNCLX #2 and shNCLX #3). The control for these experiments was HASMCs stably expressing control scrambled shRNA (shScramble). Because of the lack of reliable commercial NCLX antibodies, we measured NCLX mRNA in these cells using RT–quantitative PCR (qPCR) techniques. NCLX mRNA expression was significantly reduced in shNCLX #2 and shNCLX #3 HASMCs compared with shScramble HASMCs (Fig. 1*A*). There was no compensatory change in protein expression of MCU between NCLX KD and shScramble HASMCs (Fig. 1, *H* and *I*).

We next measured NCLX function in these HASMCs by assessing mitochondrial Ca^2+^ dynamics using the mitochondrial Ca^2+^-sensitive dye, Rhod-2 AM. As a control, MitoTracker Green was coloaded into cells. HASMCs were stimulated with 500 nM of the agonist, bradykinin, which stimulates the G protein–coupled receptor, B_2_. Activation of the B_2_ receptor induces the activation of phospholipase C, which stimulates the production of inositol-1,4,5-trisphosphate (IP_3_). Soluble IP_3_ binds and activates the IP_3_ receptors in the ER to release ER Ca^2+^. This Ca^2+^ release into the cytosol is thought to be transferred to the mitochondria through the MCU channel complex and eventually extruded from mitochondria *via* NCLX (19). Upon stimulation of HASMCs with bradykinin in the presence of 2 mM external Ca^2+^, there is a Ca^2+^ rise in the mitochondrial matrix, which represents mitochondrial Ca^2+^ uptake and subsequent decay corresponding to mitochondrial Ca^2+^ extrusion. HASMCs stably expressing either shNCLX #2 or shNCLX #3 had significantly higher levels of basal (unstimulated) mitochondrial Ca^2+^ and reduced rates of mitochondrial Ca^2+^ extrusion compared with shScramble HASMCs. Although the inhibited rate of mitochondrial Ca^2+^ extrusion did not reach statistical significance for HASMCs stably expressing shNCLX #2. Meanwhile, mitochondrial Ca^2+^ uptake was not significantly different between these three conditions (Fig. 1, *B*–*E*).

With these stable NCLX KD HASMCs, we compared SOCE using the ratiometric cytoplasmic Ca^2+^ indicator, Fura2. HASMCs were passively depleted of their ER Ca^2+^ stores using the irreversible sarcoplasmic/ER Ca^2+^ ATPase inhibitor, thapsigargin (2 μM), in a nominally Ca^2+^-free bath solution. SOCE activity was revealed upon reintroduction of 2 mM Ca^2+^ into the bath solution. SOCE was significantly reduced in shNCLX #2 and shNCLX #3 HASMCs compared with shScramble HASMCs (Fig. 1, *F* and *G*). This is consistent with previous findings, whereby NCLX activity is required for optimal SOCE activity. NCLX inhibition causes mitochondrial Ca^2+^ overload, enhanced mitochondrial oxidant production, and inhibition of SOCE through oxidation of cysteine 195 on Orai1 (32). Similar to previous findings (32), STIM1, STIM2, and Orai1 protein expression were similar between the NCLX KD and shScramble HASMCs (Fig. 1, *J*–*M*). Because of lack of specific antibodies against Orai2 and Orai3, we measured Orai2 and Orai3 mRNA expression and found them to be also comparable between these three experimental conditions (Fig. 1, *N* and *O*). These results show that NCLX function is required for optimal SOCE in HASMCs.

### NCLX is antiapoptotic and required for HASMC proliferation and migration

**Figure 2:**
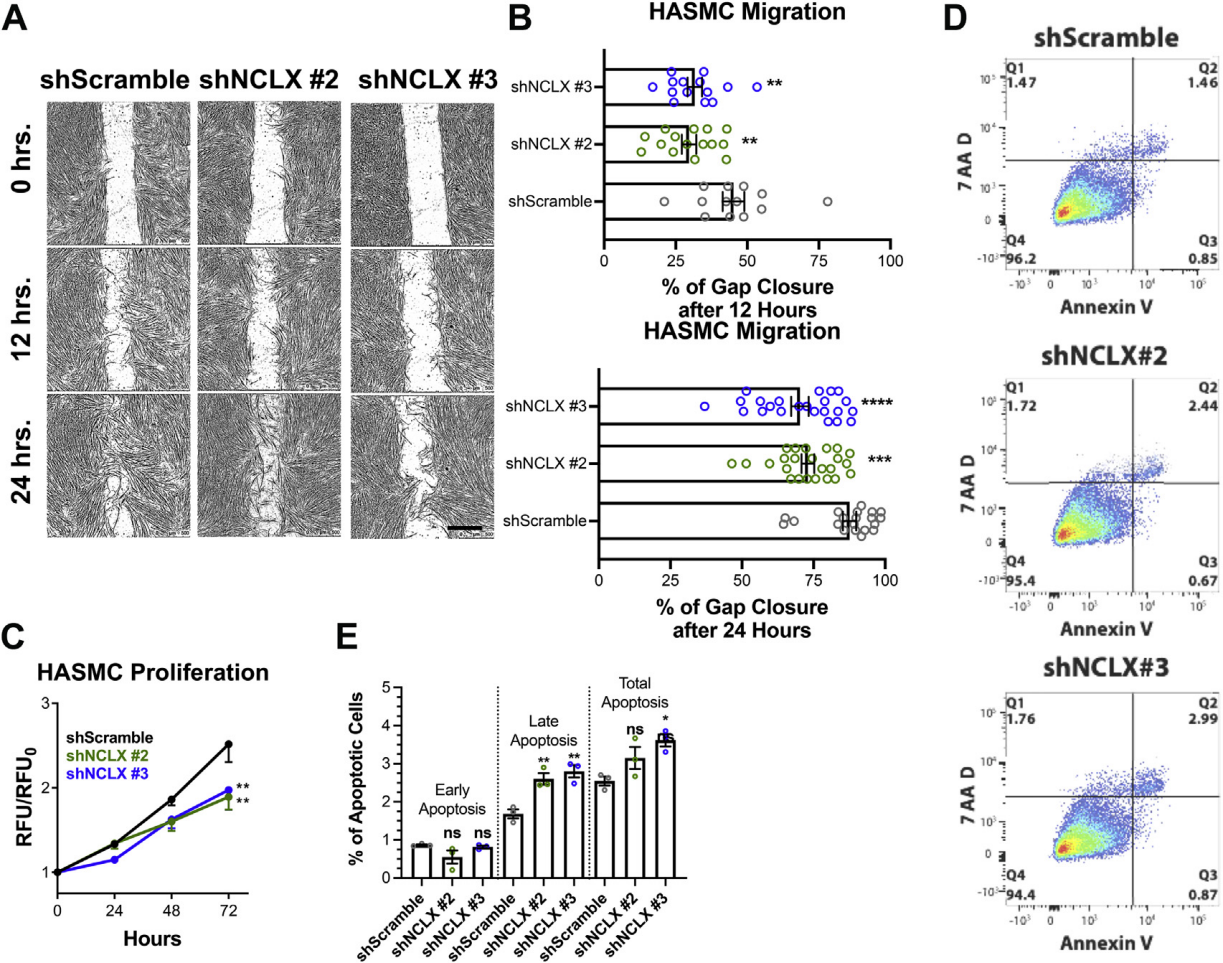
**NCLX is essential for proliferation, migration, and apoptosis in HASMCs.***A*, representative bright field images of HASMC migration at 0, 12, and 24 h in shScramble (n = 13), shNCLX #2 (n = 15), and shNCLX #3 (n = 14) HASMCs. The scale bar represents 500 μm. *B*, quantification of HASMC migration from *A* (for 12 h: one-way ANOVA, *F*[2,39] = 7.907, *p* = 0.0013, with Dunnett’s post hoc test, *p* = 0.0013 for shNCLX #2 and *p* = 0.0056 for shNCLX #3; for 24 h: one-way ANOVA, *F*[2,64] = 12.24, *p* < 0.0001, with Dunnett’s post hoc test, *p* = 0.0004 for shNCLX #2 and *p* < 0.0001 for shNCLX #3). *C*, quantification of normalized relative fluorescent units (RFUs) of proliferation from shScramble (*black trace*) (n = 6), shNCLX #2 (*green trace*) (n = 6), and shNCLX #3 (*blue trace*) (n = 6) HASMCs over 72 h (two-way ANOVA: interaction *F*[6,75] = 2.441, *p* = 0.0329; row factor *F*[3,75] = 70.00, *p* < 0.0001; column factor *F*[2,75] = 7.147, *p* = 0.0014; for Dunnett’s post hoc test, 24 h: *p* = 0.9966 for shNCLX #2 and *p* = 0.3135 for shNCLX #3, 48 h: *p* = 0.1135 for shNCLX #2 and *p* = 0.1774 for shNCLX #3, and 72 h: *p* = 0.0003 for shNCLX #2 and *p* = 0.0015 for shNCLX #3. *D*, representative flow cytometry dot plots of costaining for 7-AAD and annexin V in shScramble, shNCLX #2, and shNCLX #3 HASMCs. *E*, quantification of percent of cells in early apoptosis (quadrant 3) (one-way ANOVA, *F*[2,6] = 2.737, *p* = 0.1430, with Dunnett’s post hoc test, *p* = 0.1243 for shNCLX #2 and *p* = 0.9278 for shNCLX #3), late apoptosis (quadrant 2) (one-way ANOVA, *F*[2,6] = 17.02, *p* = 0.0034, with Dunnett’s post hoc test, *p* = 0.0075 for shNCLX #2 and *p* = 0.0028 for shNCLX #3), and total apoptosis (quadrant 2 + 3) (one-way ANOVA, *F*[2,6] = 6.971, *p* = 0.0272, with Dunnett’s post hoc test, *p* = 0.1373 for shNCLX #2 and *p* = 0.0174 for shNCLX #3) in shScramble (n = 3), shNCLX #2 (n = 3), and shNCLX #3 (n = 3) HASMCs from flow cytometry dot plots in *D*. ∗*p* < 0.05, *∗∗p* < 0.01, ∗∗∗*p* < 0.001, *∗∗∗∗p* < 0.0001, and ns. 7-AAD, 7-aminoactinomycin D; HASMC, human airway smooth muscle cell; Na^+^/Ca^2+^ exchanger; ns, not significant.

SOCE is essential for vascular and ASM proliferation, migration, and remodeling in animal models of hypertension, restenosis, and asthma (10, 11, 12, 13, 14, 16). Hence, we explored the possibility that through its regulation of SOCE, NCLX regulates ASM proliferation and migration. We measured HASMC migration using the gap closure assay where culture media were supplemented with 10 μg/ml of mitomycin C to eliminate contributions from HASMC proliferation. HASMCs transduced with shNCLX #2 and shNCLX #3 had significantly less migration compared with shScramble HASMCs at both 12 and 24 h (Fig. 2, *A* and *B*). HASMC proliferation measured over 72 h using the dye CyQUANT showed that shNCLX #2 and shNCLX #3 HASMCs proliferated significantly less than shScramble HASMCs (Fig. 2*C*). We have previously reported that NCLX KO in colorectal cancer cells inhibited proliferation and enhanced apoptosis (29). We costained shScramble, shNCLX #2, and shNCLX #3 HASMCs with 7-aminoactinomycin D (7-AAD; Tonbo Biosciences) and annexin V, which allows determination of early and late apoptosis. Annexin V binds to phosphatidylserine on the outer membrane of early apoptotic cells. 7-AAD enters necrotic and permeable cells and binds to the DNA of late apoptotic cells. HASMCs transfected with shNCLX #2 and shNCLX #3 had significantly more percentage of cells in late apoptosis (*double positive for annexin V and 7-AAD*) and total apoptosis (*sum of early and late apoptosis*). The percentage of cells in early apoptosis was unchanged (Fig. 2, *D* and *E*). These results suggest that NCLX is critical for the ASM proliferation, migration, and for preventing apoptosis.

### NCLX regulates proremodeling transcriptional reprogramming in HASMCs

**Figure 3:**
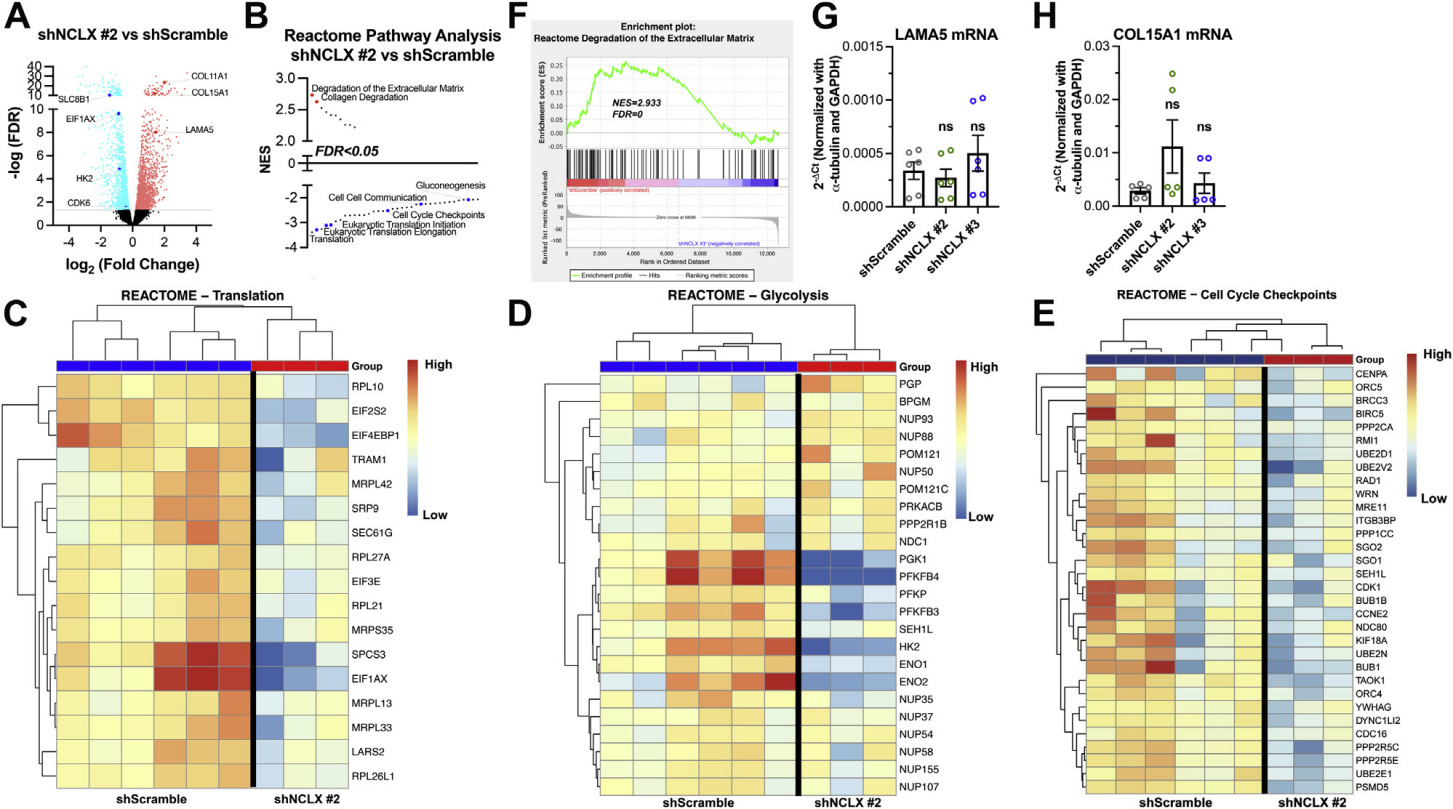
**NCLX regulates the expression of genes involved in proliferation, metabolism, and translation.***A*, volcano plot comparing differentially expressed genes between shScramble and shNCLX #2 HASMCs. *B*, pathway analysis of *A* using the Reactome pathways gene set ranking significantly upregulated (*red*) and downregulated (*blue*) pathways based on normalized enrichment score (NES). Heatmaps comparing differential expression of shScramble and shNCLX #2 HASMCs for (*C*) translation, (*D*) glycolysis, and (*E*) cell cycle checkpoints from the Reactome pathway gene sets. *F*, GSEA enrichment plots using the Reactome pathways gene set comparing shNCLX #3 and shScramble HASMCs showing a positive correlation in the enrichment of degradation of the extracellular matrix. Quantification of (*G*) LAMA5 (one-way ANOVA, *F*[2,15] = 1.024, *p* = 0.3829, with Dunnett’s post hoc test, *p* = 0.8873 for shNCLX #2 and *p* = 0.5273 for shNCLX #3) and (*H*) COL15A1 (one-way ANOVA, *F*[2,12] = 2.069, *p* = 0.1690, with Dunnett’s post hoc test, *p* = 0.1409 for shNCLX #2 and *p* = 0.9244 for shNCLX #3) mRNA expression in shScramble (n = 5), shNCLX #2 (n = 5), and shNCLX #3 (n = 5) HASMCs using RT–quantitative PCR. GSEA, gene set enrichment analysis; HASMC, human airway smooth muscle cell; NCLX, Na^+^/Ca^2+^ exchanger; ns, not significant.

**Figure 4:**
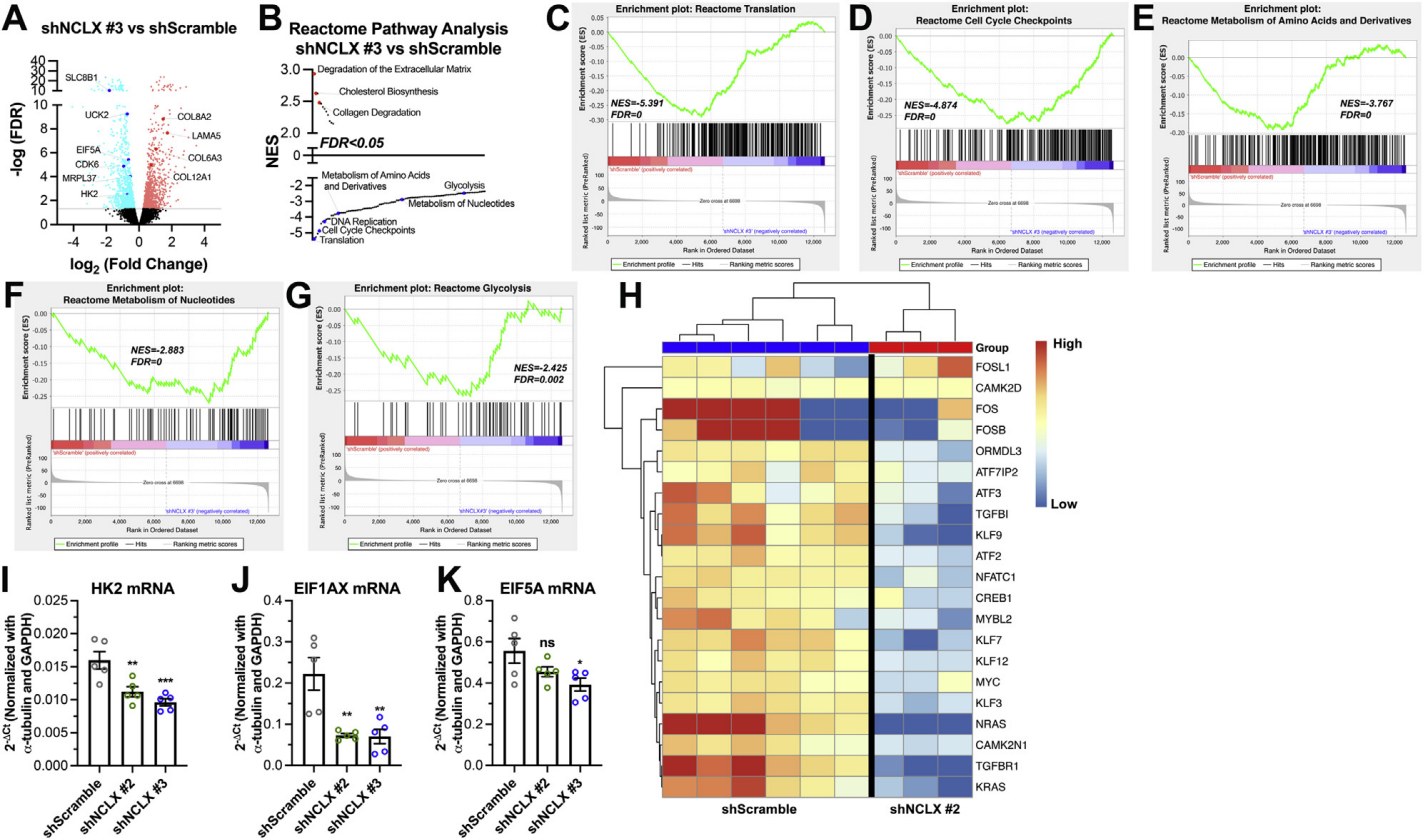
**NCLX regulates the expression of transcription factors and metabolic genes.***A*, volcano plot comparing differentially expressed genes between shScramble and shNCLX #3 HASMCs. Genes are plotted by log_2_ fold change and −log_10_ false discovery rate (FDR), with a threshold of an FDR <0.05 (*dotted black line*). In the shNCLX #3 samples, significantly upregulated and downregulated genes are *light red* and *light blue*, respectively. *B*, pathway analysis of *A* using the Reactome pathways gene set ranking significantly upregulated (*red*) and downregulated (*blue*) pathways based on normalized enrichment score (NES). *C*–*H*, GSEA enrichment plots using the Reactome pathways gene set comparing shNCLX #3 and shScramble HASMCs showing a negative correlation in the enrichment of (*C*) translation, (*D*) cell cycle checkpoints, (*E*) metabolism of amino acids and derivatives, (*F*) metabolism of nucleotides, and (*G*) glycolysis. For each GSEA enrichment plot, NES and FDR values are displayed. *H*, heatmap comparing expression of select genes from RNA sequencing of shScramble and shNCLX#2 HASMCs. Quantification of (*I*) HK2 (one-way ANOVA, *F*[2,12] = 12.51, *p* = 0.0012, with Dunnett’s post hoc test, *p* = 0.0067 for shNCLX #2 and *p* = 0.0008 for shNCLX #3), (*J*) EIF1AX (one-way ANOVA, *F*[2,12] = 12.00, *p* = 0.0014, with Dunnett’s post hoc test, *p* = 0.0023 for shNCLX #2 and *p* = 0.0020 for shNCLX #3), (*K*) and EIF5A (one-way ANOVA, *F*[2,12] = 3.952, *p* = 0.0480, with Dunnett’s post hoc test, *p* = 0.1895 for shNCLX #2 and *p* = 0.0300 for shNCLX #3), mRNA expression in shScramble (n = 5), shNCLX #2 (n = 5), and shNCLX #3 (n = 5) HASMCs using RT–quantitative PCR. ∗*p* < 0.05, ∗∗*p* < 0.01, *∗∗∗p* < 0.001, and ns. GSEA, gene set enrichment analysis; HASMC, human airway smooth muscle cell; HK2, hexokinase 2; NCLX, Na^+^/Ca^2+^ exchanger; ns, not significant.

To understand the mechanism by which NCLX regulates HASMC function and identify potential downstream pathways regulated by NCLX in HASMCs, we compared the transcriptional profiling of shScramble, shNCLX #2, and shNCLX #3 HASMCs using RNA-Seq. We found many genes that were differently expressed between shScramble *versus* shNCLX #2 (Fig. 3*A*) or shNCLX #3 HASMCs (Fig. 4*A*). Using gene set enrichment analysis (GSEA) and the Reactome gene set, we observed numerous enriched pathways between shScramble *versus* shNCLX #2 (Fig. 3*B*) or shNCLX #3 HASMCs (Fig. 4*B*). Negatively enriched pathways in shNCLX #2 and shNCLX #3 include translation, cell cycle checkpoints, metabolism of amino acids and derivatives, metabolism of nucleotides, and glycolysis (Figs. 3, *C*–*E* and 4, *C*–*G*). Positively enriched pathways included degradation of the extracellular matrix (Fig. 3*F*). Interestingly, numerous Ca^2+^-dependent transcription factors like c-FOS, nuclear factor of activated T cell (NFATC1), and cAMP response element–binding protein (CREB1) as well as the asthma-associated protein ORMDL3 were downregulated in the NCLX KD HASMCs (Fig. 4*H*). Through qPCR, we validated some of these RNA-Seq findings and found shNCLX #2 and shNCLX #3 HASMCs have a significant decrease in hexokinase 2, EIF1AX, and EIF5A mRNA expression compared with shScramble HASMCs (Fig. 4,*I*–*K*). However, the expression of LAMA5 and COL15A1, which were hits in the degradation of the extracellular matrix GSEA pathway analysis, was not significantly different between experimental groups (Fig. 3, *A*, *G*,and *H*). Altogether, these RNA-Seq findings suggest that NCLX is essential for maintaining signaling pathways that support a dedifferentiated proliferative migratory phenotype in HASMCs. While a number of these pathways have been associated specifically with SOCE in smooth muscle (16, 17), additional mechanisms specifically related to mitochondria are clearly involved. These mechanisms are explored further.

### NCLX deficiency in HASMCs induces mitochondrial depolarization, mitophagy, inhibits CaMKII phosphorylation, and quells metabolic activity

**Figure 5:**
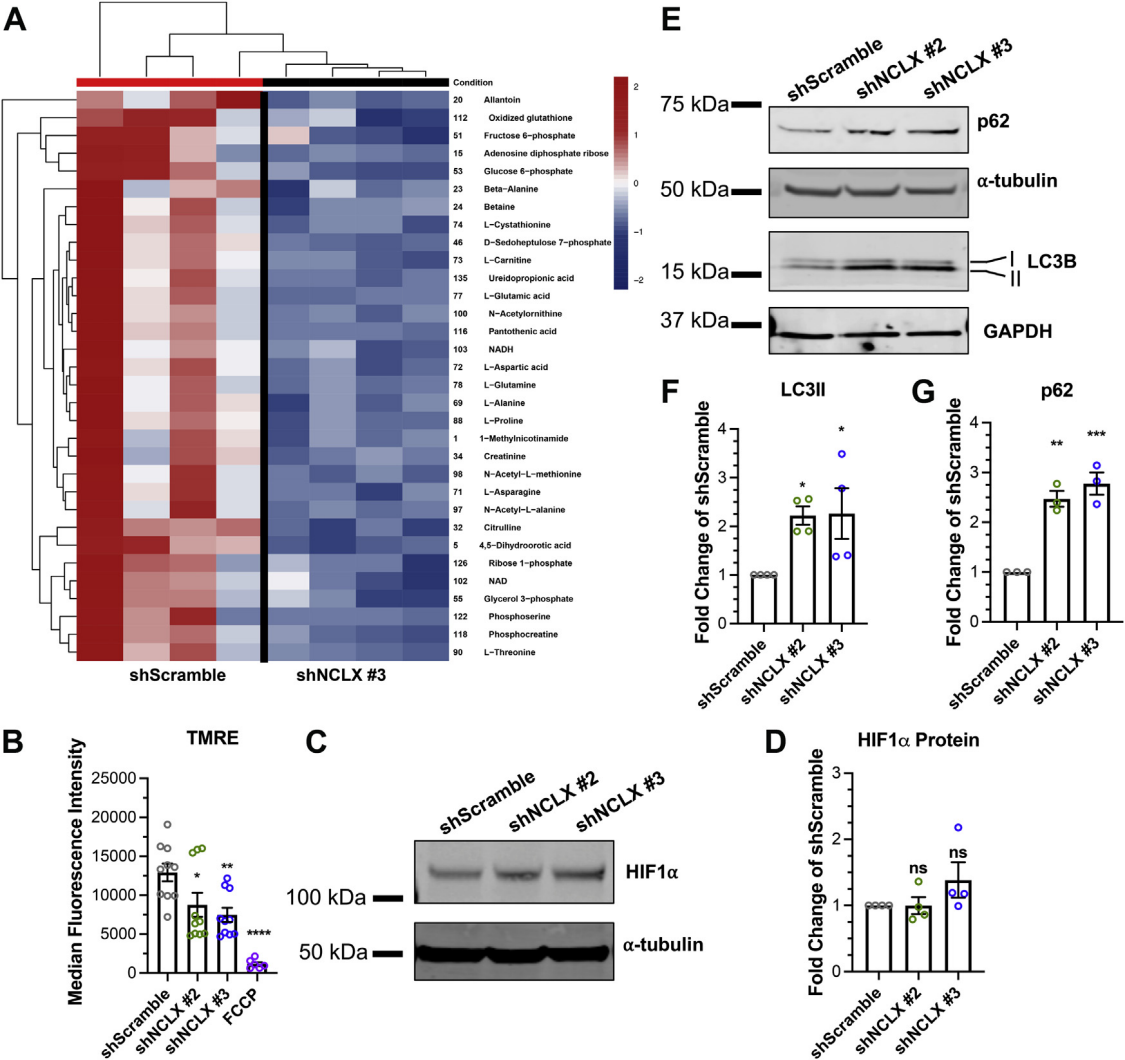
**Abrogation of NCLX enhances mitophagy in HASMCs.***A*, heatmap comparing abundance of statistically significant metabolites in shScramble and shNCLX #3 HASMCs. *B*, quantification of TMRE (membrane potential dye) median fluorescence intensity (MFI) of shScramble (n = 10), shNCLX #2 (n = 10), shNCLX #3 (n = 10), shScramble + 100 μM FCCP (n = 6) in HASMCs using flow cytometry (one-way ANOVA, *F*[3,32] = 13.70, *p* < 0.0001, with Dunnett’s post hoc test, *p* = 0.0374 for shNCLX #2, *p* = 0.0051 for shNCLX #3, and *p* < 0.0001 for FCCP). *C*, representative Western blot showing HIF1α protein expression in shScramble, shNCLX #2, and shNCLX #3 HASMCs. (Note that the α-tubulin blot for Figure 1*H* was reused for the α-tubulin blot in Figure 5*C*. The molecular weights for MCU and HIF1α were far enough to allow cutting and separate immunoblotting from the same blot). *D*, quantification of HIF1α (one-way ANOVA, *F*[2,9] = 1.67, *p* = 0.2406, with Dunnett’s post hoc test, *p* > 0.9999 for shNCLX #2 and *p* = 0.2471 for shNCLX #3) protein expression from *C* in shScramble (n = 4), shNCLX #2 (n = 4), and shNCLX #3 (n = 4) HASMCs using densitometry normalized to α-tubulin. *E*, representative Western blot showing p62 and LC3 protein expression in shScramble, shNCLX #2, and shNCLX #3 HASMCs. Quantification of (*F*) LC3II (one-way ANOVA, *F*[2,9] = 5.023, *p* = 0.0343, with Dunnett’s post hoc test, *p* = 0.0436 for shNCLX #2 and *p* = 0.0378 for shNCLX #3) and (*G*) p62 (one-way ANOVA, *F*[2,6] = 35.72, *p* = 0.0005, with Dunnett’s post hoc test, *p* = 0.0011 for shNCLX #2 and *p* = 0.0004 for shNCLX #3) protein expression from *A* in shScramble (n = 3), shNCLX #2 (n = 3), and shNCLX #3 (n = 3) HASMCs using densitometry normalized to GAPDH and α-tubulin. ∗*p* < 0.05, ∗*∗p* < 0.01, ∗∗∗*p* < 0.001, *∗∗∗∗p* < 0.0001, and ns. FCCP, carbonyl cyanide-4-(trifluoromethoxy)phenylhydrazone; HASMC, human airway smooth muscle cell; HIF1α, hypoxia-inducible factor 1α; LC3II, light chain 3II; MCU, mitochondrial Ca^2+^ uniporter; NCLX, Na^+^/Ca^2+^ exchanger; ns, not significant; TMRE, tetramethylrhodamine, ethyl ester.

An interesting finding from our transcriptional profiling and GSEA analyses was that numerous metabolic pathways were downregulated in the NCLX KD HASMCs. This included pathways for the metabolism of amino acids and derivatives, metabolism of nucleotides, and glycolysis (Figs. 3*D* and 4, *E*–*G*), in agreement with a recent report showing that NCLX was essential for metabolic activity of colorectal cancer cells (29). Previous reports have showed that ASM cells from bronchi of asthmatic patients have enhanced metabolism and mitochondrial mass (16, 26, 27), prompting speculation that this enhanced metabolism provided the necessary energy for ASM proliferation and growth. Therefore, we used metabolomics and found that numerous metabolites in glycolysis, citric acid (TCA) cycle, and amino acid metabolism were significantly downregulated in shNCLX #3 compared with shScramble HASMCs (Fig. 5*A*). These results indicate that abrogation of NCLX reduces glycolysis, TCA, and amino acid metabolism and that mitochondria are likely dysfunctional in shNCLX HASMCs. Then, we explored the possibility that mitochondria are depolarized in shNCLX HASMCs. We and others have showed that NCLX KO or KD in a number of cell types causes mitochondrial Ca^2+^ overload (28, 29, 36, 37), which induces the activation of the mPTP and depolarizes the mitochondria. We used the dye, tetramethylrhodamine, ethyl ester (TMRE), as an indicator of mitochondrial membrane potential. We found that shNCLX #2 and shNCLX #3 HASMCs have significantly less TMRE staining compared with shScramble HASMCs, suggesting that the mitochondria of NCLX KD HASMCs were significantly depolarized (Fig. 5*B*). As a positive control, the ionophore carbonyl cyanide-4-(trifluoromethoxy)phenylhydrazone (FCCP) completely abolished TMRE staining. Previous reports have found PM potential to also affect TMRE staining (38, 39). However, FCCP greatly inhibited TMRE fluorescence suggesting that most of the TMRE staining is mitochondrial. We also explored the protein expression of the transcription factor, hypoxia-inducible factor 1α, which is known to be induced by mitochondrial dysfunction. However, hypoxia-inducible factor 1α protein expression was marginally increased in shNCLX #2 and shNCLX #3 compared with shScramble HASMCs, and this increase was not statistically significant (Fig. 5, *C* and *D*).

**Figure 6:**
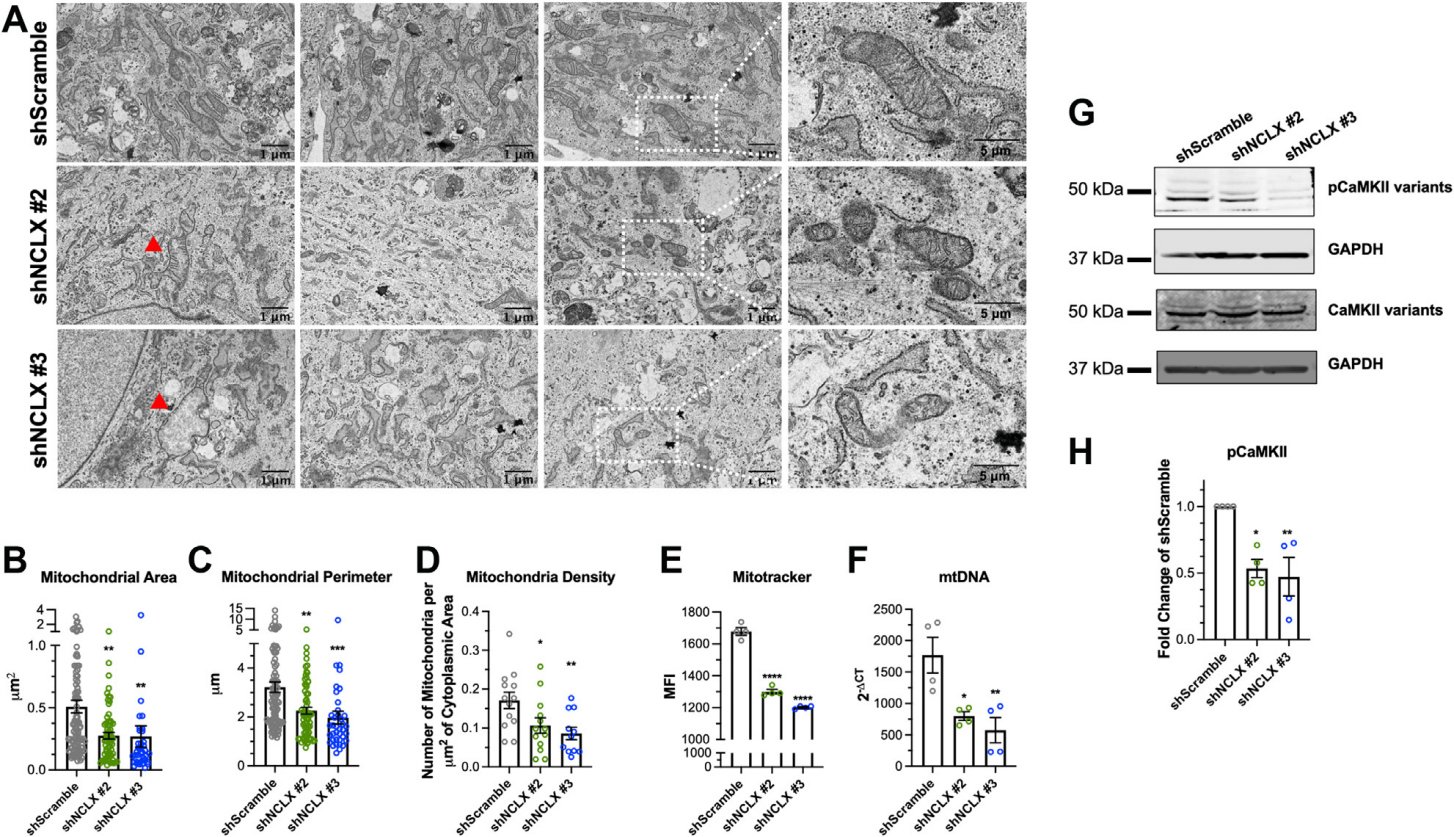
**Loss of NCLX leads to reduced size and number of mitochondria in HASMCs.***A*, representative transmission electron microcopy (TEM) images of shScramble, shNCLX #2, and shNCLX #3 HASMCs. Last images on *right* are zoomed insets. (The scale bar represents 1 and 5 μm for larger images and zoomed insets, respectively). *Red arrows* point to mitochondria with altered structure. Using TEM, quantification of the (*B*) mitochondrial area (one-way ANOVA, *F*[2199] = 7.330, *p* = 0.0008, with Dunnett’s post hoc test, *p* = 0.0020 for shNCLX #2 and *p* = 0.0087 for shNCLX #3), and (*C*) mitochondrial perimeter (one-way ANOVA, *F*[2199] = 9.871, *p* < 0.0001, with Dunnett’s post hoc test, *p* = 0.0013 for shNCLX #2 and *p* = 0.0004 for shNCLX #3), from shScramble (n = 99), shNCLX #2 (n = 65), and shNCLX #3 (n = 38) HASMCs. *D*, quantification of the mitochondrial density or number of mitochondria per μm^2^ of cytoplasmic area of micrographs from shScramble (n = 13), shNCLX #2 (n = 13), and shNCLX #3 (n = 11) HASMCs (one-way ANOVA, *F*[2,34] = 5.175, *p* = 0.0109, with Dunnett’s post hoc test, *p* = 0.0399 for shNCLX #2 and *p* = 0.0090 for shNCLX #3). *E*, quantification of median fluorescence intensity (MFI) of MitoTracker *Green* FM in shScramble (n = 4), shNCLX #2 (n = 4), and shNCLX #3 (n = 4) HASMCs with using flow cytometry (one-way ANOVA, *F*[2,9] = 238.6, *p* < 0.0001, with Dunnett’s post hoc test, *p* < 0.0001 for shNCLX #2 and *p* < 0.0001 for shNCLX #3). *F*, quantification of mitochondrial (mtDNA) in shScramble (n = 4), shNCLX #2 (n = 4), and shNCLX #3 (n = 4) HASMCs using RT–qPCR. mtDNA CT values are normalized to CT values of genomic DNA products (one-way ANOVA, *F*[2,9] = 9.648, *p* = 0.0058, with Dunnett’s post hoc test, *p* = 0.0154 for shNCLX #2 and *p* = 0.0047 for shNCLX #3). *G*, representative Western blots showing phosphorylated CaMKII variants (pCAMKII) and total CAMKII variant protein expression in HASMCs transfected with shScramble and two NCLX shRNA (shNCLX #2 and shNCLX #3). *H*, quantification of pCAMKII protein in shScramble (n = 4), shNCLX #2 (n = 4), and shNCLX #3 (n = 4) HASMCs from *G* using densitometry normalized to total CAMKII and GAPDH (one-way ANOVA, *F*[2,9] = 9.829, *p* = 0.0055, with Dunnett’s post hoc test, *p* = 0.0109 for shNCLX #2 and *p* = 0.0053 for shNCLX #3). ∗p < 0.05, ∗∗*p* < 0.01, ∗∗*∗p* < 0.001, *∗∗∗∗p* < 0.0001, ns, not significant.

Depolarization of mitochondria often leads to the induction of mitophagy. Mitophagy is the regulated removal of mitochondria through autophagy and is believed to be a mechanism of mitochondrial quality control. The autophagy marker, microtubule-associated protein 1A/1B-light chain 3B (LC3B), undergoes cleavage from a cytosolic LCBI form to a smaller LC3BII form that becomes associated with autophagic vesicles. HASMCs transduced with shNCLX #2 and shNCLX #3 had significantly more LC3BII expression than shScramble HASMCs (Fig. 5, *E* and *F*). Furthermore, the downstream autophagy receptor, p62, was also significantly increased in shNCLX #2 and shNCLX #3 HASMCs compared with shScramble HASMCs (Fig. 5, *E* and *G*). We utilized transmission electron microscopy and observed an enhanced proportion of mitochondria in shNCLX #2 and shNCLX#3 HASMCs that are swollen or have collapsed cristae structures (Fig. 6*A*). Interestingly, the remaining mitochondria in the shNCLX #2 and shNCLX #3 HASMCs had significantly smaller areas and perimeters compared with the mitochondria of shScramble HASMCs (Fig. 6, *B* and *C*). In addition, mitochondrial density was significantly decreased in the shNCLX #2 and shNCLX #3 HASMCs (Fig. 6*D*). We used flow cytometry and MitoTracker Green staining and found that HASMCs transfected with shNCLX #2 and shNCLX #3 had significantly less MitoTracker staining than shScramble HASMCs (Fig. 6*E*), indicating reduced number of functional mitochondria. We also measured mitochondrial DNA (mtDNA) using qPCR techniques; shNCLX #2 and shNCLX #3 HASMCs had less mtDNA than shScramble HASMCs (Fig. 6*F*). Overall, these results suggest that NCLX KD HASMCs have increased mitophagy with fewer mitochondria.

The Ca^2+^ effector protein, CaMKII, is a crucial signaling molecule activated by local increases of Ca^2+^ through Ca^2+^–calmodulin complexes, which bind to multimers of CaMKII and induce autophosphorylation of the complex (40). The most abundant CaMKII isoform in synthetic VSMC is the δ isoform, which has been shown to be necessary for proliferation, migration, and neointima formation (41, 42). Specifically, CaMKII phosphorylates and activates numerous proteins as well as stimulates transcriptional reprogramming in VSMCs. However, very little is known regarding the role of CaMKII in ASM, except for a report showing that CaMKIIδ protein is increased in ASM of asthmatic mice and necessary for AHR in asthma (43). Recent studies also suggest that CaMKII compartmentalizes with mitochondria in VSMCs and cardiomyocytes (44, 45, 46, 47). Therefore, we investigated whether NCLX regulates the phosphorylation and activation of CaMKII. We utilized a monoclonal antibody that recognizes the autophosphorylation of threonine 286 on CaMKII variants. HASMCs transduced with shNCLX #2 and shNCLX #3 had significantly less pCaMKII than shScramble HASMCs (Fig. 6, *G* and *H*). This suggests that NCLX is required for the activation of CaMKII and that subsequent downstream signaling from CaMKII is most likely ablated in shNCLX HASMCs.

### ASM NCLX is necessary for AR, fibrosis, and hyperresponsiveness in asthmatic mice

**Figure 7:**
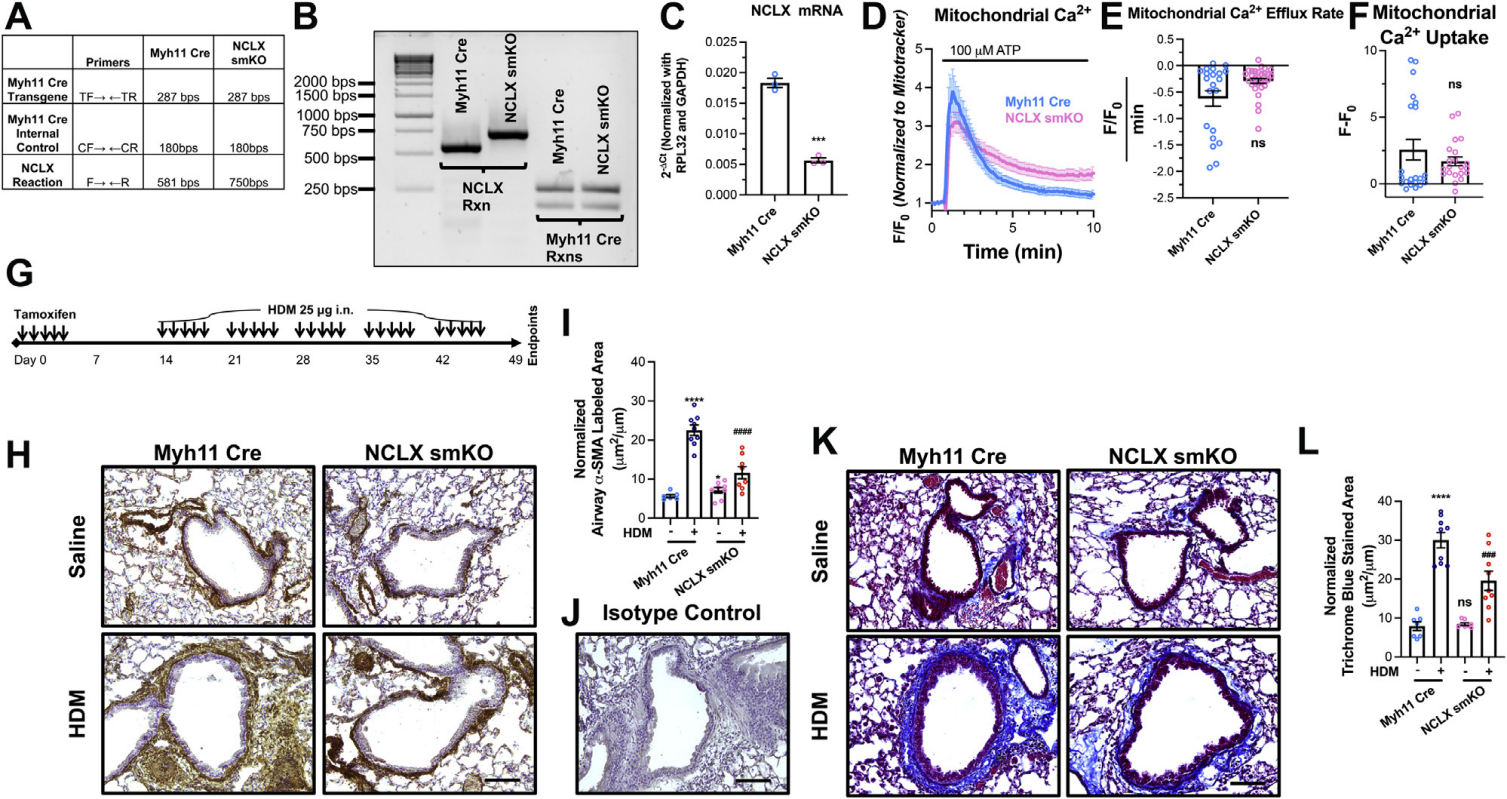
**ASM NCLX is necessary for airway remodeling (AR) in a mouse model of house dust mite (HDM)–induced asthma.***A*, table of PCR products for NCLX and Myh11 Cre from Myh11 Cre and NCLX smKO mice. Table lists predicted band sizes of each PCR in each mouse cohort. See supporting appendix for sequence of primers. *B*, gel showing PCR products from genotyping reactions in *A* from the tails of Myh11 Cre and NCLX smKO mice. *C*, quantification of NCLX mRNA expression in isolated mouse ASM cells (MASMCs) from Myh11 Cre (n = 3) and NCLX smKO (n = 3) mice using RT–qPCR (Student’s *t* test, *p* = 0.0001). *D*, mitochondrial Ca^2+^ measurements stimulated with 100 μM ATP in the presence of 2 mM Ca^2+^ showing mitochondrial Ca^2+^ influx and extrusion in Myh11 Cre (*light blue trace*) (n = 21) and NCLX smKO (*pink trace*) (n = 30) cultured MASMCs. Mitochondrial Ca^2+^ is measured by dividing the fluorescence of Rhod-2 by that of MitoTracker *Green*. Quantification of (*E*) mitochondrial Ca^2+^ efflux rate (Mann–Whitney test, *p* = 0.3565) and (*F*) mitochondrial Ca^2+^ uptake (Mann–Whitney test, *p* = 0.3302) from *D*. *G*, scheme illustrating the timeline of tamoxifen injections and intranasal HDM challenges. *H*, representative immunohistochemistry images of lung slices from Myh11 Cre and NCLX smKO mice challenged with either saline or HDM. Smooth muscle is labeled *brown* with α-SMA antibody and 3,3′-diaminobenzidine (DAB). Slides are also counterstained with hematoxylin. The scale bar represents 100 μm. *I*, quantification of α-SMA-labeled area from *H* in saline-challenged Myh11 Cre (n = 6), HDM-challenged Myh11 Cre (n = 9), saline-challenged NCLX smKO (n = 8), and HDM-challenged NCLX smKO (n = 8) mice (two-way ANOVA: interaction *F*[1,27] = 79.71, *p* < 0.0001; genotype factor *F*[1,27] = 15.38, *p* = 0.0005; challenge factor *F*[1,27] = 26.75, *p* < 0.0001; for Turkey’s post hoc test compared with saline-challenged Myh11 Cre: *p* < 0.0001 for HDM-challenged Myh11 Cre, *p* = 0.0166 for saline-challenged NCLX smKO, *p* = 0.8347 for HDM-challenged NCLX smKO; compared with HDM-challenged Myh11 Cre: *p* < 0.0001 for HDM-challenged NCLX smKO). *J*, lung slices labeled with an isotype control and stained with DAB for immunohistochemistry. Lung slices were counterstained with hematoxylin. The scale bar represents 100 μm. *K*, representative images of lung slices stained with Masson’s trichrome stain. Collagen is stained *blue*. The scale bar represents 100 μm. *L*, quantification of *blue*-stained area from *C* in saline-challenged Myh11 Cre (n = 7), HDM-challenged Myh11 Cre (n = 9), saline-challenged NCLX smKO (n = 8), and HDM-challenged NCLX smKO (n = 9) mice (two-way ANOVA: interaction *F*[1,29] = 9.107, *p* = 0.0053; genotype factor *F*[1,29] = 7.43, *p* = 0.0108; challenge factor *F*[1,29] = 83.64, *p* < 0.0001; for Turkey’s post hoc test compared with saline-challenged Myh11 Cre: *p* < 0.0001 for HDM-challenged Myh11 Cre, *p* = 0.9972 for saline-challenged NCLX smKO, *p* = 0.0006 for HDM-challenged NCLX smKO; compared with HDM-challenged Myh11 Cre: *p* < 0.0007 for HDM-challenged NCLX smKO). *∗p* < 0.05, *∗∗p* < 0.01, ∗∗∗*∗p* < 0.0001, ns, not significant when compared with saline-challenged Myh11 Cre. *###p* < 0.001, *####p* < 0.0001 when compared with HDM-challenged Myh11 Cre. ASM, airway smooth muscle; NCLX, Na^+^/Ca^2+^ exchanger; qPCR, quantitative PCR; α-SMA, α-smooth muscle actin.

With these mechanistic insights (*i.e.*, decreased proliferation, migration, transcriptional reprogramming, activation of CaMKII, and metabolic dysfunction), we tested whether these *in vitro* findings translate to an *in vivo* mouse model. We generated NCLX smKO mice by crossing the current gold-standard tamoxifen-inducible smooth muscle Cre (Myh11 Cre), with mice containing NCLX loxP-flanked alleles (NCLX^fl/fl^) (28). Genotyping of the tracheas from NCLX smKO and Myh11 Cre littermate mice confirms that both mice have the transgenic Myh11 Cre, and the NCLX smKO mice have loxP-flanked alleles (Fig. 7, *A* and *B*). To confirm that NCLX is knocked out, we isolated mouse ASM cells (MASMCs) from epithelium-denuded tracheas of Myh11 Cre and NCLX smKO mice. Using RT–qPCR, we measured NCLX mRNA and found that it was significantly reduced in NCLX smKO MASMCs compared with Myh11 Cre MASMCs (Fig. 7*C*). In addition, we also measured NCLX function in MASMCs loaded with the mitochondrial dye Rhod-2. MASMCs were stimulated with 100 μM of the agonist ATP, which stimulates P2Y receptors, in the presence of 2 mM extracellular Ca^2+^. NCLX smKO MASMCs showed slower mitochondrial Ca^2+^ efflux rate compared with Myh11 Cre MASMCs (Fig. 7, *D* and *E*), although this did not reach statistical significance. Mitochondrial Ca^2+^ uptake was also not statistically different between NCLX smKO and Myh11 Cre MASMCs (Fig. 7, *D* and *F*).

We then intranasally sensitized and challenged Myh11 Cre and NCLX smKO mice to the allergen, house dust mite (HDM) (Fig. 7*G*). Chronic high exposures to HDM induce an asthmatic phenotype in mice with AR, fibrosis, and hyperresponsiveness. The control conditions for these experiments were Myh11 Cre and NCLX smKO mice challenged with saline. Following this asthma protocol, we isolated, fixed, and labeled lung slices with α-smooth muscle actin (α-SMA) to image smooth muscle remodeling through immunohistochemistry techniques. As previously reported, HDM-challenged Myh11 Cre mice had much more α-SMA-labeled area (*brown in images*) than saline-challenged Myh11 Cre mice. Strikingly, HDM-challenged NCLX smKO mice had dramatically less airway α-SMA-labeled area than the HDM-challenged Myh11 Cre mice. Both saline-challenged mice had similar amounts of α-SMA-labeled area (Fig. 7,*H* and *I*). The isotype control had no immunohistochemistry staining for α-SMA (Fig. 7*J*). To examine airway fibrosis, we stained these cohorts with Masson’s trichrome stain, which stains collagen and extracellular matrix fibers blue. HDM-challenged Myh11 Cre mice have dramatically more Trichrome blue–stained area than the saline-challenged Myh11 Cre mice. Interestingly, the HDM-challenged NCLX smKO mice had significantly less stained area than the HDM-challenged Myh11 Cre mice. Saline-challenged NCLX smKO mice also had similar amounts of Masson’s trichrome staining as the saline-challenged Myh11 Cre mice (Fig. 7, *K* and *L*).

**Figure 8:**
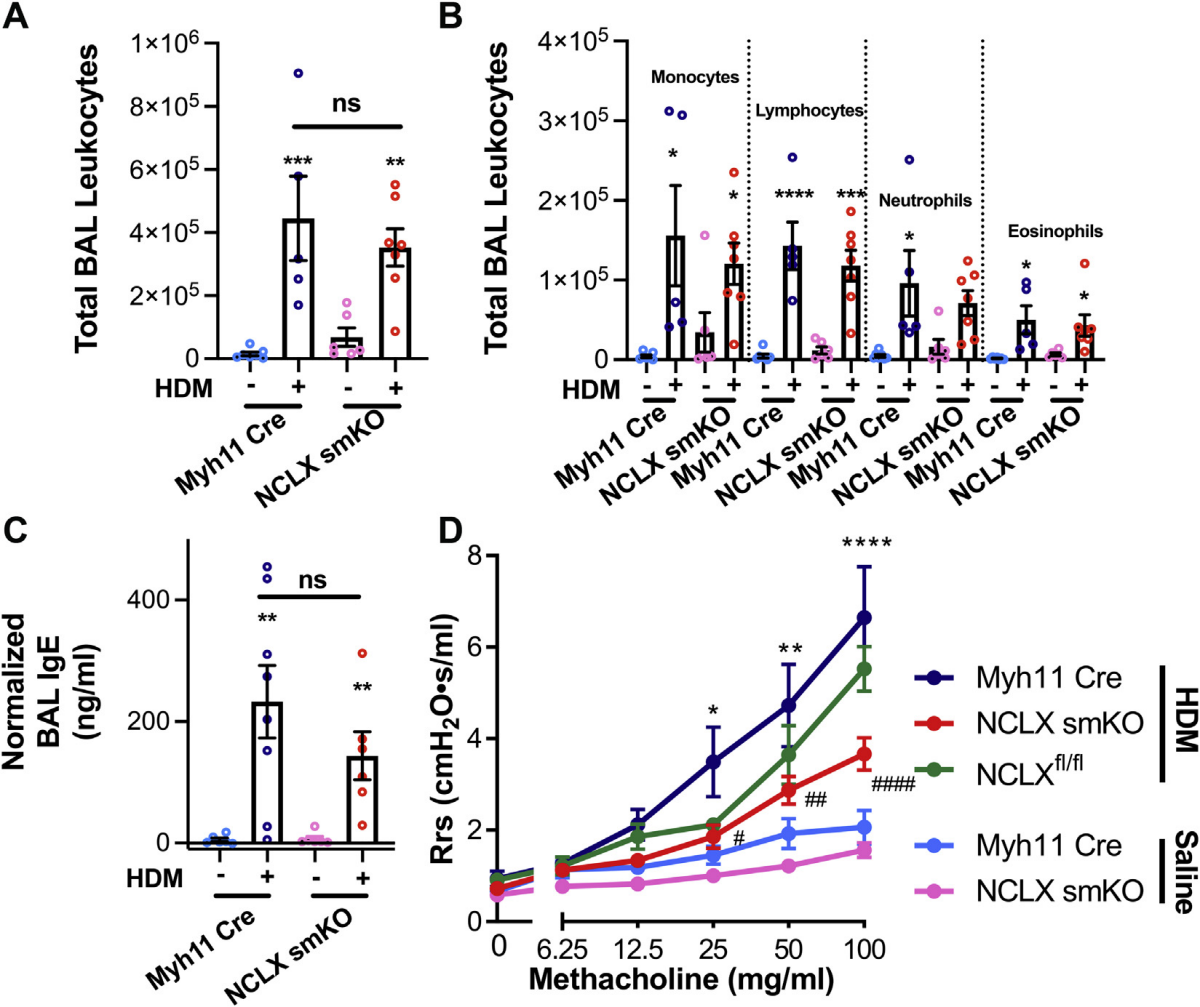
**ASM NCLX is necessary for airway hyperresponsiveness (AHR) in a mouse model of house dust mite (HDM)–induced asthma.***A*, quantification of the total number of bronchoalveolar lavage (BAL) leukocytes (two-way ANOVA: interaction *F*[1,21] = 1.278, *p* = 0.270; genotype factor *F*[1,21] = 0.09157, *p* = 0.7652; challenge factor *F*[1,21] = 30.77, *p* < 0.0001; for Turkey’s post hoc test compared with saline-challenged Myh11 Cre: *p* = 0.0008 for HDM-challenged Myh11 Cre, *p* = 0.9360 for saline-challenged NCLX smKO, *p* = 0.0037 for HDM-challenged NCLX smKO; compared with HDM-challenged Myh11 Cre: *p* = 0.7575 for HDM-challenged NCLX smKO) and (*B*) different leukocyte subsets including monocytes (two-way ANOVA: interaction *F*[1,21] = 1.090, *p* = 0.3083; genotype factor *F*[1,21] = 0.007221, *p* = 0.9331; challenge factor *F*[1,21] = 14.38, *p* = 0.0011; for Turkey’s post hoc test compared with saline-challenged Myh11 Cre: *p* = 0.153 for HDM-challenged Myh11 Cre, *p* = 0.8974 for saline-challenged NCLX smKO, *p* = 0.0486 for HDM-challenged NCLX smKO; compared with HDM-challenged Myh11 Cre: *p* = 0.8632 for HDM-challenged NCLX smKO), lymphocytes (two-way ANOVA: interaction *F*[1,21] = 0.9436 *p* = 0.3424; genotype factor *F*[1,21] = 0.2990, *p* = 0.5903; challenge factor *F*[1,21] = 72.19, *p* < 0.0001; for Turkey’s post hoc test compared with saline-challenged Myh11 Cre: *p* < 0.0001 for HDM-challenged Myh11 Cre, *p* = 0.9895 for saline-challenged NCLX smKO, *p* = 0.0002 for HDM-challenged NCLX smKO; compared with HDM-challenged Myh11 Cre: *p* = 0.7243 for HDM-challenged NCLX smKO), neutrophils (two-way ANOVA: interaction *F*[1,21] = 0.9021, *p* = 0.3530; genotype factor *F*[1,21] = 0.1266, *p* = 0.7256; challenge factor *F*[1,21] = 14.72, *p* = 0.0010; for Turkey’s post hoc test compared with saline-challenged Myh11 Cre: *p* = 0.0165 for HDM-challenged Myh11 Cre, *p* = 0.9724 for saline-challenged NCLX smKO, *p* = 0.0688 for HDM-challenged NCLX smKO; compared with HDM-challenged Myh11 Cre: *p* = 0.8047 for HDM-challenged NCLX smKO), and eosinophils (two-way ANOVA: interaction *F*[1,21] = 0.3289, *p* = 0.5724; genotype factor *F*[1,21] = 0.01048, *p* = 0.9194; challenge factor *F*[1,21] = 16.47, *p* = 0.0006; for Turkey’s post hoc test compared with saline-challenged Myh11 Cre: *p* = 0.0210 for HDM-challenged Myh11 Cre, *p* = 0.9859 for saline-challenged NCLX smKO, *p* = 0.0327 for HDM-challenged NCLX smKO; compared with HDM-challenged Myh11 Cre: *p* = 0.9656 for HDM-challenged NCLX smKO) from saline-challenged Myh11 Cre (n = 7), HDM-challenged Myh11 Cre (n = 5), saline-challenged NCLX smKO (n = 6), and HDM-challenged NCLX smKO mice (n = 7). *C*, quantification of IgE in the BAL of saline-challenged Myh11 Cre (n = 6), HDM-challenged Myh11 Cre (n = 8), saline-challenged NCLX smKO (n = 6), and HDM-challenged NCLX smKO (n = 6) mice (two-way ANOVA: interaction *F*[1,21] = 0.6326, *p* = 0.4353; genotype factor *F*[1,21] = 0.5819, *p* = 0.4541; challenge factor *F*[1,21] = 20.65, *p* = 0.0002; for Turkey’s post hoc test compared with saline-challenged Myh11 Cre: *p* = 0.0033 for HDM-challenged Myh11 Cre, *p* > 0.9999 for saline-challenged NCLX smKO, *p* = 0.0035 for HDM-challenged NCLX smKO; compared with HDM-challenged Myh11 Cre: *p* = 0.6882 for HDM-challenged NCLX smKO). For each sample in *C*, IgE secretion is normalized to total protein content in sample. *D*, trace measuring airway resistance (Rrs) on anesthetized mice challenged with increasing concentrations of methacholine. Mice cohorts are saline-challenged Myh11 Cre (*light blue trace*) (n = 12), HDM-challenged Myh11 Cre (*dark blue trace*) (n = 12), HDM-challenged NCLX^fl/fl^ mice (*green trace*) (n = 5), saline-challenged NCLX smKO (*pink trace*) (n = 10), and HDM-challenged NCLX smKO (*red trace*) (n = 12) mice (two-way ANOVA: interaction *F*[24,303] = 3.997, *p* < 0.0001; *row factor F*[6303] = 38.84, *p* < 0.0001; column factor *F*[6303] = 26.57, *p* = 0.0014; for Dunnett’s post hoc test compared with saline-challenged Myh11 Cre 0 mg/ml of methacholine: *p* = 0.9603 for HDM-challenged Myh11 Cre, *p* = 0.9997 for saline-challenged NCLX smKO, *p* = 0.9998 for HDM-challenged NCLX smKO, *p* = 0.9896 for HDM-challenged NCLX^fl/fl^; compared with HDM-challenged Myh11 Cre 0 mg/ml of methacholine: *p* = 0.9580 for HDM-challenged NCLX smKO; compared with saline-challenged Myh11 Cre 6.25 mg/ml of methacholine: *p* = 0.9971 for HDM-challenged Myh11 Cre, *p* = 0.9053 for saline-challenged NCLX smKO, *p* ≥ 0.9999 for HDM-challenged NCLX smKO, *p* = 0.9998 for HDM-challenged NCLX^fl/fl^; compared with HDM-challenged Myh11 Cre 6.25 mg/ml of methacholine: *p* = 0.9850 for HDM-challenged NCLX smKO; compared with saline-challenged Myh11 Cre 12.5 mg/ml of methacholine: *p* = 0.2095 for HDM-challenged Myh11 Cre, *p* = 0.9129 for saline-challenged NCLX smKO, *p* = 0.9953 for HDM-challenged NCLX smKO, *p* = 0.7154 for HDM-challenged NCLX^fl/fl^; compared with HDM-challenged Myh11 Cre 12.5 mg/ml of methacholine: *p* = 0.3140 for HDM-challenged NCLX smKO; compared with saline-challenged Myh11 Cre 25 mg/ml of methacholine: *p* = 0.0003 for HDM-challenged Myh11 Cre, *p* = 0.8349 for saline-challenged NCLX smKO, *p* = 0.8645 for HDM-challenged NCLX smKO, *p* = 0.7270 for HDM-challenged NCLX^fl/fl^; compared with HDM-challenged Myh11 Cre 25 mg/ml of methacholine: *p* = 0.0071 for HDM-challenged NCLX smKO; compared with saline-challenged Myh11 Cre 50 mg/ml of methacholine: *p* < 0.0001 for HDM-challenged Myh11 Cre, *p* = 0.5309 for saline-challenged NCLX smKO, *p* = 0.247 for HDM-challenged NCLX smKO, *p* = 0.0419 for HDM-challenged NCLX^fl/fl^; compared with HDM-challenged Myh11 Cre 50 mg/ml of methacholine: *p* = 0.0018 for HDM-challenged NCLX smKO; compared with saline-challenged Myh11 Cre 100 mg/ml of methacholine: *p* < 0.0001 for HDM-challenged Myh11 Cre, *p* = 0.8066 for saline-challenged NCLX smKO, *p* = 0.0134 for HDM-challenged NCLX smKO, *p* < 0.0001 for HDM-challenged NCLX^fl/fl^; compared with HDM-challenged Myh11 Cre 100 mg/ml of methacholine: *p* < 0.0001 for HDM-challenged NCLX smKO. ∗*p* < 0.05, *∗∗p* < 0.01, ∗∗∗*p* < 0.001, ∗∗*∗∗p* < 0.0001, ns, not significant when compared with saline-challenged Myh11 Cre. #*p* < 0.05, *##p* < 0.01, *###p* < 0.001, *####p* < 0.0001 when compared with HDM-challenged Myh11 Cre. ASM, airway smooth muscle; NCLX, Na^+^/Ca^2+^ exchanger; IgE, immunoglobulin E.

Airway inflammation and leukocyte recruitment is another hallmark feature of asthma. We measured leukocyte recruitment to the airways by collecting the bronchoalveolar lavage (BAL) from mice. HDM-challenged Myh11 Cre mice had dramatically more BAL leukocytes than saline-challenged Myh11 Cre mice (Fig. 8*A*). The subsets of these leukocytes included monocytes, lymphocytes, neutrophils, and eosinophils (Fig. 8*B*). Intriguingly, the total number and proportions of BAL leukocytes were similar between HDM-challenged NCLX smKO and HDM-challenged Myh11 Cre mice (Fig. 8, *A* and *B*). The saline-challenged cohorts also had similar BAL leukocytes (Fig. 8, *A* and *B*). We also measured BAL immunoglobin E (IgE). IgE is predominantly secreted by plasma cells. The HDM-challenged Myh11 Cre mice had significantly more IgE than saline-challenged STIM1 smKO mice (Fig. 8*C*). Importantly, while HDM-challenged NCLX smKO mice had lower amounts of IgE as compared with HDM-challenged Myh11 Cre mice, this difference was not statistically significant. IgE levels were also similar between the saline-challenged cohorts (Fig. 8*C*). These results indicate that ASM NCLX is intrinsically critical for the development of AR, including smooth muscle hyperplasia and airway fibrosis and that this effect is independent of leukocyte recruitment to the airways.

AHR is due to excessive contraction of asthmatic airways in response to stimuli and is another cardinal feature of asthma. We measured AHR to methacholine (MeCh) in the chronic HDM-challenged asthmatic mice using the small animal ventilator, Flexivent. HDM-challenged Myh11 Cre had significantly more AHR than saline-challenged Myh11 Cre mice, as documented by increased airway resistance (Rrs) in response to increasing doses of MeCh (Fig. 8*D*). For these experiments, we included another important cohort of mice as a control, which corresponds to NCLX^fl/fl^ expressing Myh11 Cre mice but injected with a vehicle control instead of tamoxifen (referred to as NCLX^fl/fl^ mice). When these NCLX^fl/fl^ mice were HDM challenged, they showed a similar extent of AHR as HDM-challenged Myh11 Cre mice. Strikingly, the HDM-challenged NCLX smKO mice had significantly less AHR than the HDM-challenged Myh11 Cre or NCLX^fl/fl^ mice (Fig. 8*D*).

## Discussion

Here, we identified a novel function for the NCLX, in regulating ASM proliferation and migration in both *in vitro* and *in vivo* models of AR in asthma. We have previously reported that NCLX regulates SOCE and Ca^2+^ release–activated Ca^2+^ current in VSMCs (32), and we and others have also shown that smooth muscle SOCE is critical for driving airway and vascular remodeling (10, 11, 12, 13, 14, 15). SOCE activates fibroproliferative programs through transcription factors like NFAT, CREB, and c-Fos. In light of our recent findings describing a key function for STIM1 in AR and AHR of asthmatic mice (16), our data herein suggest that NCLX function contributes to AR, at least partially through maintaining optimal SOCE activity in ASM.

Mitochondrial Ca^2+^ is not completely ablated in the shNCLX HASMCs. Similar findings were reported in colorectal cancer cells, astrocytes, and cardiomyocytes (28, 29, 30), suggesting that NCLX is not the sole mechanism for mitochondrial Ca^2+^ extrusion. Interestingly, Rysted *et al.* (48) studied isolated mitochondria from different tissues and reported that while NCLX contributes to Ca^2+^ extrusion from brain and heart mitochondria, it plays a marginal role, if any, in liver mitochondria. Other mechanisms like mitochondrial Ca^2+^ extrusion potentially include leucine zipper and EF-hand containing transmembrane protein 1 (LETM1) and mPTP (49, 50, 51). Therefore, the mechanisms of mitochondrial Ca^2+^ extrusion are likely cell type–specific and would require further clarification. Currently, the only commercially available inhibitor of NCLX is CGP-37157, which has numerous off-target effects (51, 52). Another challenge is the subcellular localization of exogenously expressed tagged NCLX, which was shown to localize in other organelles beside mitochondria, including intracellular vesicles, PM, and the ER (48, 53, 54). There is a lack of specific antibodies against NCLX, and the localization of the native NCLX protein in different cell types requires clarification.

We also discovered that NCLX is required for the activation of CaMKII. We identified the phosphorylation of CaMKII to be dependent on NCLX. CaMKII is a multifunctional kinase that phosphorylates numerous targets. This includes channels like MCU, IP_3_ receptors, voltage-gated Ca^2+^ channels, and sarcoplasmic/ER Ca^2+^ ATPase as well as transcription factors including activating factor-1, myocyte elongation factor 2, and NF-κB (55, 56). Hence, it is a major hub for Ca^2+^ signaling and serves to activate numerous fibroproliferative pathways. However, additional studies are required to identify, which of these downstream pathways are regulated by NCLX in ASM. Future studies should examine how NCLX precisely regulates CaMKII phosphorylation and which specific CAMKII species becomes phosphorylated. Studies have reported the existence of a pool of CaMKII associated with mitochondria (44, 45, 46, 47). Such pool may directly sense Ca^2+^ at the vicinity of NCLX. Another possibility is that NCLX is indirectly regulating CaMKII through SOCE.

Strikingly, our transcriptional profiling experiments revealed numerous signaling pathways that were differentially expressed in NCLX KD HASMCs compared with control cells. The top hit, protein translation, was significantly downregulated in NCLX KD HASMCs. Multiple signaling molecules like mammalian target of rapamycin, tuberous sclerosis protein (TSC), and Ca^2+^ itself have been described to regulate protein synthesis. Other top pathways include cell cycle checkpoints, DNA replication, and numerous metabolic pathways. The mechanisms of how NCLX regulates these pathways most likely consist of a combination of Ca^2+^ signaling coupled to SOCE microdomains, CaMKII activation, and induction of metabolic programs. We also showed that NCLX is critical in the regulation of ASM metabolism. Downregulation of NCLX leads to reduced levels of amino acids and nucleotides, reduced mitochondria number and structure, and enhanced mitophagy. These data are consistent with our previously reported findings where NCLX was necessary for metabolic activity of colorectal cancer cells (29).

Using an *in vivo* mouse asthma model, we showed that NCLX smKO mice have less AR, consistent with the described function of NCLX in ASM proliferation, migration through the mechanisms described previously (metabolic and transcriptional reprogramming). Interestingly, we also observed that NCLX smKO mice have less airway fibrosis. Although the molecular mechanisms of airway fibrosis are still unclear, ASM cells have emerged as significant immunomodulatory cells in the airways capable of secreting cytokines (*e.g.*, interleukin 6 [IL-6]) and extracellular matrix (16, 57). Previous studies showed that SOCE is critical for IL-6 secretion by airway epithelial cells and that the asthma-inducing allergen HDM mediates SOCE-dependent secretion of IL-6 by airway epithelial cells (58, 59, 60). We recently showed that STIM1 is required for the secretion of IL-6 by ASM from asthmatic mice through Ca^2+^-dependent activation of NFAT4 (16). It is therefore likely that NCLX regulates the secretion of extracellular matrix and cytokines such as IL-6 by ASM cells, partially through SOCE, CaMKII, and/or other mechanisms.

We also demonstrated that NCLX smKO exposed to the allergen HDM have significantly less AHR. Although a cardinal feature of asthma, the molecular mechanisms of AHR are still unclear. Certain studies suggest that AR directly contributes to AHR arguing that hypertrophic and hyperplastic ASM would increase airway resistance (61). However, other studies suggest AR and AHR are independent (62, 63). Overexpression of certain structural genes in mice has also been associated with asthma, AR, and AHR in the absence of airway inflammation (64, 65). NCLX might be regulating AHR through both AR and the aforementioned mechanisms, including SOCE, CaMKII, and metabolism. STIM1 and SOCE-mediated Ca^2+^ signals are essential for AHR (16), and whether NCLX regulates AHR, at least partially, through promoting SOCE or directly through mitochondrial Ca^2+^ efflux or through other means is an interesting question that requires further investigations.

In summary, we have unveiled a novel and complex role for NCLX in ASM remodeling during asthma. NCLX promotes ASM proliferation, migration, and inhibits apoptosis through a multifaceted mechanism that likely include SOCE, CaMKII, transcriptional reprogramming, mitochondrial Ca^2+^, and metabolism. This complex mechanism emphasizes the critical role NCLX plays in both Ca^2+^ signaling and mitochondrial homeostasis in ASM. Although previous efforts to target Ca^2+^ influx signals with L-type Ca^2+^ channel blockers and Ca^2+^ chelators have failed (66, 67), it is possible these therapies did not specifically target discrete Ca^2+^ signaling microdomains that promotes AR and AHR. Thus, NCLX may be a novel target for the treatment of asthma.

## Experimental procedures

### Cell culture and stable KD cells

HASMCs were isolated and cultured from human tracheas received from the National Disease Research Interchange and the International Institute for the Advancement of Medicine. Since the tracheas are derived from deidentified donors, research using HASMCs is not classified as human subject research by Penn State’s and Rutgers’s Institutional Review Board. HASMCs are derived from donors without any pulmonary disease and died from other causes. HASMCs were cultured in Ham’s F-12 media supplemented with 1% l-glutamine (Gibco), 1% penicillin/streptomycin (Gibco), 0.2% Primocin (InvivoGen), and 10% fetal bovine serum (Hyclone) at 37 °C, 5% CO_2_, and 100% humidity as previously described (33). Serum-free media include no fetal bovine serum, but it is instead supplemented with 1% bovine serum albumin. All HASMC experiments were completed between passages 2 and 5.

The shNCLX #2 and shNCLX #3 pLKO.1 lentiviral shRNA constructs were obtained from the Broad’s Institute’s RNAi Consortium Library at Penn State’s Library at the Penn State’s Genome Science core (see supporting methods for sequences). Lentiviruses were generated by packaging shScramble, shNCLX #2, and shNCLX #3 pLKO.1 lentiviral constructs into human embryonic kidney 293FT using the ViraPower kit (Invitrogen) and Lipofectamine 2000 (Thermo Fisher Scientific). After infecting HASMCs with lentiviruses for 72 h, cells were selected with 2.5 μg/ml of puromycin for an additional 72 h. To confirm KD, qPCR and mitochondrial Ca^2+^ imaging techniques were utilized. Stable KD HASMCs were used within 2 weeks for experiments.

### qPCR

Total RNA was isolated and quantified using the RNAeasy Minikit (Qiagen) and the nanodrop 2000 spectrophotometer (Thermo Fisher Scientific). Using the High Capacity cDNA Reverse Transcription Kit (Applied Biosystems), 1 μg of total RNA was transcribed into complementary DNA (cDNA). SYBR Green qPCR Master Mix (Applied Biosystems), corresponding primers (see supporting table), and cDNA were loaded into a 96-well plate. Using the QuantStudio 3 real-time PCR system (Applied Biosystems), target mRNA expression was recorded through the following PCR protocol: 50 °C 2-min activation step, a 95 °C 2-min melt step, and 40 cycles of 95 °C for 15 s followed by 57 °C for 15 s and 72 °C for 30 s. For each target, melt curves were generated and analyzed. Target mRNA expression was quantified by comparative C_t_ method and normalized to housekeeping genes. qPCR experiments were performed in technical and biological triplicates.

### Western blotting

Cells at 80 to 90% confluency were washed with ice-cold PBS and lysed with radioimmunoprecipitation assay lysis buffer (MilliporeSigma) supplemented with fresh protease and phosphatase inhibitors (Thermo Fisher Scientific). About 30 μg of protein lysate was then loaded into a 4 to 12% Bis–Tris gel (Thermo Fisher Scientific), and following gel electrophoresis, gels were transferred onto a polyvinylidene difluoride membrane (MilliporeSigma). After membranes were blocked with Olympus Blocking Buffer (LI-COR) for 1 h at room temperature, blocked membranes were incubated overnight with primary antibodies diluted in Olympus Blocking Buffer and 0.1% Tween (see supporting methods for dilutions and sources of antibodies). Membranes were then washed three times with 0.1% Tris-buffered saline with Tween-20 (TBST) for 5 min each and incubated in secondary antibody (1:10,000 dilution; 680RD conjugated antimouse [LI-COR] and 1:5000 dilution; 800CW conjugated anti-rabbit [LI-COR]) diluted in Olympus Blocking Buffer with 0.1% Tween for 1 h at room temperature. Following three additional washes with 0.1% TBST for 5 min each, membranes were imaged using Odyssey Clx imaging system. Densitometry was quantified using ImageStudio Lite software (LI-COR). Each densitometry quantification was normalized to the expression of a housekeeping gene (*i.e.*, α-tubulin, GAPDH).

### Single-cell cytosolic and mitochondrial Ca^2+^ imaging

To measure cytosolic Ca^2+^, HASMCs were seeded onto 25 mm glass coverslips for 24 h in complete media. Coverslips were mounted into an Attofluor cell chamber (Thermo Fisher Scientific) and incubated with 4 μM of Fura2-AM (Thermo Fisher Scientific) and 0.1% Pluronic F-127 (Thermo Fisher Scientific) in complete media for 45 min. Using Hepes-buffered salt solution (HBSS) containing 140 mM NaCl, 4.7 mM KCl, 1.13 mM MgCl_2_, 10 mM Hepes, 2.0 mM CaCl_2_, and 10 mM glucose with a pH adjusted to 7.4 by NaOH, HASMCs were washed four times, and on last wash, cells were incubated in HBSS for 10 min at room temperature. Chambers were then mounted to a Leica DMi8 Fluorescent Microscope equipped with a 20x Fluor objective. Using a faster shutter wheel (Sutter Instruments), Fura 2 was alternatively excited at 340 and 380 nm, and corresponding emissions were collected at 510 nm. The ratio of fluorescence at 340 and 380 (F_340_/F_380_) was recorded for each pixel using a Hamamatsu Flash 4 camera. Regions of interest (ROIs) were drawn around the perimeter of each cell, and the F_340_/F_380_ was analyzed for 15 to 35 cells per coverslip.

For measuring mitochondrial Ca^2+^, HASMCs were seeded onto 25 mm glass coverslips for 24 h in complete media. The coverslips were then mounted into an Attofluor cell chamber and incubated with 1.5 μM Rhod-2 AM and 50 nM of MitoTracker Green in serum-free media for 30 min at room temperature. Cells were then washed 4× with HBSS, and in last wash, cells were left in HBSS for 10 min. The chambers were then mounted to a Leica DiM8 confocal microscope with a 63× objective. MitoTracker Green was excited with a 488 nm laser, and the corresponding emission was captured with a GFP filter. Rhod-2 was excited with a 543 nm laser, and the corresponding emission was captured at 580 to 650 nm. Using Lecia Applicate Suite X software, unbiased ROIs were drawn around the mitochondria of cells. About 5 to 10 ROIs were drawn for each coverslip. Rhod-2 and MitoTracker fluorescence intensity was normalized to the fluorescence at time 0, and to correct any changes in focus, Rhod-2 fluorescence was normalized to MitoTracker fluorescence. Mitochondrial Ca^2+^ uptake was measured by subtracting maximum fluorescence at *t* = 1.4 min by fluorescence at *t* = 0. Mitochondrial Ca^2+^ efflux was calculated by calculating the slope of fluorescence between maximum uptake (*t* = 1.4 min) to *t* = 4 min.

### HASMC proliferation and migration assays

Migration was measured by seeding 20,000 HASMCs into silicon inserts with a 500 μm gap in a 6-well culture plate for 24 h. Cells were then starved in serum-free media for 24 h to synchronize cells. After removing inserts, complete media supplemented with 10 μg/ml of mitomycin C (Sigma–Aldrich) were placed on HASMCs. Bright field images were captured at 0, 12, and 24 h using a Leica DMi8 Fluorescent Microscope equipped with a 4× objective. Percent gap closure was calculated by normalizing the size of the gap area between 0 and 12 or 24 h by the area at 0 h.

To measure proliferation, 2000 HASMCs were seeded in each well of a 96-well tissue culture plate. For time 0 recording, three wells from each condition were then washed with PBS and stained with CyQUANT Cell Proliferation Assay (Thermo Fisher Scientific) dye for 1 h at 37 °C, 5% CO_2_, and 100% humidity after 1-h postseeding. A plate reader (Flexstation 3) was used to record fluorescence from these wells at 485 and 530 nm excitation and emission, respectively. The background fluorescence was subtracted from all readings. Plates were then returned to 37 °C, 5% CO_2_, and 100% humidity for further readings at 24, 48, and 72 h. Readings were normalized to time 0 recording and represented as relative fluorescent unit/relative fluorescent unit_0_.

### Flow cytometry

To measure HASMC apoptosis, cells were costained for FITC–annexin V (Tonbo Biosciences) and 7-AAD. For each sample, 200,000 HASMCs were harvested and washed once with cold PBS. The cells were then resuspended with 100 μl of annexin V buffer (10 mM Hepes, 150 mM NaCl, and 2.5 mM CaCl_2_ in PBS with a pH adjusted to 7.4) and 5 μl of 7-AAD and 5 μl of FITC–annexin V. After incubating samples for 30 min at room temperature, 400 μl of annexin V buffer was added, and data were collected on LSRII flow cytometer (BD Biosciences) and further analyzed by FlowJo software (BD Biosciences).

Mitochondrial membrane potential was measured by staining 200,000 HASMCs per sample with 150 nM of TMRE, perchlorate (Thermo Fisher Scientific) diluted in complete media. Samples were incubated for 45 min at 37 °C, 5% CO_2_, and 100% humidity. As a control, some samples were stimulated with 100 μM of FCCP during incubation. Following incubation with TMRE, samples were washed with 500 μl of fluorescence-activated cell sorting buffer. Data were collected on LSRII flow cytometer and further analyzed by FlowJo software.

To measure mitochondrial mass, 200,000 HASMCs per sample were stained with 50 nM of MitoTracker (Thermo Fisher Scientific) diluted in HBSS for 20 min in 37 °C non-CO_2_ incubator. Samples were washed one time with HBSS, and data were collected on LSRII flow cytometer and further analyzed by FlowJo software.

### Transcriptome sequencing (RNA-Seq) and differential expression analysis

RNA was isolated and quantified using RNAeasy Minikit and the nanodrop 2000 spectrophotometer. Using 1 μg of RNA, a NEBNext Ultra 2 RNA Library Prep Kit for Illumina (catalog no.: NEB #E7775; New England Biolabs) was used to construct cDNA by Novogene following the manufacturer’s protocol. Briefly, oligo(dT) beads and two rounds of purification were used to enrich mRNA. Using fragmentation buffer, mRNA was randomly fragmented. The first-strand cDNA was synthesized using random hexamers primer, and the second strand was generated with a custom second-strand synthesis buffer (Illumina), dNTPs, RNase H, and DNA polymerase I (ds cDNA). Double-stranded cDNA library was ready after a series of terminal repair, polyadenylation, sequencing adaptor ligation, size selection, and PCR enrichment. Size distribution was analyzed using an Agilent 2100 Bioanalyzer (Agilent Technologies). The 250 to 350 bp insert libraries were quantified and sequenced on an Illumina NovaSeq 6000 Platform (Illumina) using a paired-end 150 run (2 × 150 bases). Each library generated 20 M raw reads. Using STAR (version 2.5) program (DNASTAR) (68), alignments were parsed. The counts were generated using htseq-count with default parameters, and fragments per kilobase million of each gene were calculated using the length of the gene and reads count mapped to this gene (69).

Following exploratory analyses using the gene-level read count data, lowly expressed genes were removed from the dataset. Based on the read counts, a SeqExpressionSet was produced using the EDASeq R package (70), and upper quantile normalization was applied. The RUVSeq R package (71) was then applied using k = 1 and a set of 11 housekeeping genes described by Eisenberg and Levanon (72). This identified factors of unwanted variation were included as a covariate in the differential expression analysis that was subsequently performed with edgeR (Bioconductor) (73, 74). Differentially expressed genes were chosen based on a false discovery rate threshold of *q* < 0.05.

### GSEA

GSEA software was used to perform a GSEA preranked analysis based on edgeR output (75). The genes in the edgeR output were ordered using a signed version of the likelihood ratio test statistic in which the sign (positive or negative) was obtained from the log fold change. The Reactome gene sets from the Molecular Signature database (https://www.gsea-msigdb.org/gsea/msigdb/index.jsp) were used to identify affected pathways.

### Metabolomics

After changing 90% confluent HASMCs with fresh media for 2 h, cells were trypsinized and washed with ice-cold PBS. The pellets were then flash frozen, and metabolites were extracted by NYU Metabolomics Core Resource Laboratory following previously described methods (76). Briefly, metablotis were first scaled to cell counts to ratio of 1 × 10^6^ cells/ml of extraction solvent (80% LC–MS grade methanol [aq] with 500 nM labeled amino acid internal standard [Cambridge Isotope Laboratories, Inc; catalog no.: MSK-A2-1.2]). Samples were homogenized with zirconium disruption beads (0.5 mm; RPI) for 5 min at 4 °C in a BeadBlaster with a 30 s on, 30 s off pattern. Following centrifugation at 21,000*g* for 3 min, 450 μl of supernatant was transferred for speed vacuum concentration. Samples were transferred to a glass insert for further analysis.

The LC column used for LC–MS/MS analysis was Millipore ZIC-pHILIC (2.1 × 150 mm, 5 μm) coupled to a Dionex ULtiMate 3000 system. The column oven temperature was set to 25 °C for the gradient elution. A flow rate of 100 μl/min was used with the following buffers: (A) 10 mM ammonium carbonate in water, pH 9.0; (B) neat acetonitrile. The gradient profile was as follows: 80 to 20% B (0–30 min), 20 to 80% B (30–31 min), and 80 to 80% B (31–42 min). Injection volume was set to 2 μl for all analyses (42 min total run time per injection). LC system coupled to a Thermo Q Exactive HF mass spectrometer operating in heated electrospray ionization mode was used to measure MS. The method duration was 30 min with a polarity switching data-dependent top 5 method for both positive and negative modes. The spray voltage for both positive and negative modes was 3.5 kV, and capillary temperature was set to 32 °C with a sheath gas rate of 35, auxiliary gas of 10, and max spray current of 100 μA. Full MS scan for both polarities was utilized at 120,000 resolution with an automatic gain control (AGC) target of 3e6 and a maximum injection time of 100 ms, and the scan range was from 67 to 1000 *m*/*z*. Tandem MS spectra for both positive and negative modes used a resolution of 15,000, AGC target of 1e5, maximum injection time of 50 ms, isolation window of 0.4 *m/z*, isolation offset of 0.1 *m/z*, fixed first mass of 50 *m/z*, and 3-way multiplexed normalized collision energies of 10, 35, and 80. The minimum AGC target was 1 × 10^4^ with an intensity of 2 × 10^5^. Data were acquired in profile mode.

Metabolomic data were processed through Python and R libraries. Peak height intensities were extracted by the established accurate mass and retention time for each metabolite (77) and verified with authentic standards and/or high-resolution MS–MS manually curated against the NIST14MS/MS (78) and METLIN (79) spectral libraries. The theoretical *m/z* of the metabolite molecular ion was used with a ±10 ppm mass tolerance window and a ±0.2 min peak apex retention time tolerance within the expected elution window (1–2 min). The median mass accuracy *versus* the theoretical *m/z* for the library was −0.6 ppm (n = 121 detected metabolites). Median retention time range (time between earliest and latest eluting sample for a given metabolite) was 0.25 min (30 min LC–MS method). A signal-to-noise ratio of 3× was used compared with blank controls throughout the sequence to report detection, with a floor of 10,000 (arbitrary units). Labeled amino acid internal standards in each sample were used to assess instrument performance (median CV% = 12%).

### mtDNA measurements

As previously described (80, 81), HASMCs were lysed and digested using DirectPCR Lysis Reagent (Viagen Biotech) and proteinase K (Viagen Biotech). After the sequential additions of isopropanol and 70% ethanol, total DNA was collected from each sample. Target mtDNA was recorded for each sample by using SYBR Green qPCR Master Mix, three sets of primers that specifically amplify regions of mtDNA, and two sets of primers that specifically amplify regions of genomic DNA (see supporting methods for sequence of primers). The PCR protocol was a 50 °C 2-min activation step, a 95 °C 2-min melt step, and 40 cycles of 95 °C for 15 s followed by 56 °C for 15 s and 72 °C for 30 s. Melt curves were generated and analyzed for each target. mtDNA copy number was quantified by comparative C_t_ method and normalized to genomic DNA.

### Transmission electron microscopy sample preparation and morphological analysis

HASMCs were grown to 90% confluency and fixed in half-strength Karnovsky fixative containing 2% paraformaldehyde and 2.5% glutaraldehyde (pH 7.3). Samples were fixed again in 1% osmium tetroxide in 0.1 M Sorenson's buffer (pH 7.4) for 30 min. The samples were then dehydrated in a graduated ethanol series, and they were embedded in LX-112 (Ladd Research). About 60 nm sections were stained with uranyl acetate and lead citrate. Samples were viewed in a JEOL JEM1400 Transmission Electron Microscope (JEOL USA, Inc). Using ImageJ software (NIH), the morphology and density of mitochondria was analyzed from at least 10 different micrographs of each sample. Analysis was blinded. Mitochondrial density was calculated by counting the number of mitochondria in the micrograph and normalizing to the area of cytoplasm. Morphological parameters (*i.e.*, aspect ratio, circularity, roundness, and solidity) was calculated by manually tracing the outer mitochondrial membrane.

### Mouse models

Institutional Animal Care and Use Committee approved all animal studies. NCLX^fl/fl^ (28) and Myh11 Cre (82) mice have been previously described. All mice are on a pure C57BL/6. All experimental mice were genotyped through tail tips and PCRs with primers specific for each transgenic mouse (see supporting methods for primer sequences). To induce activation of Myh11 Cre, 6- to 7-week-old mice were intraperitonially injected with 100 μl of tamoxifen (MilliporeSigma) dissolved in sunflower oil (10 mg/ml) daily for 5 consecutive days. After 1 week, mice were ready for further experiments.

The asthmatic mouse model was induced in male NCLX smKO and littermate controls (NCLX^fl/fl^ and Myh11 Cre). These cohorts were first anesthetized with isoflurane and then intranasally challenged with 25 μg of HDM (*Dermatphagoides pteronyssinus*) (Greer Laboratories, Inc) suspended in 40 μl of PBS at 8 to 9 weeks of age. At same time, controls were challenged with PBS alone. All cohorts were challenged for 5 consecutive days followed with 2 days of rest for 5 weeks. After 72 h of last challenge, mice were measured for AHR and tissue/BAL were collected.

### Measurement of AHR to MeCh

After NCLX smKO and littermate controls were aged for 13 to 14 weeks, they were anesthetized with 7 mg/ml of pentobarbital intraperitoneally. Cohorts were then intubated and ventilated using a tracheal catheter and a small animal ventilator, flexiVent FX1 (SCIEQ). Dynamic airway resistance was measured when mice were nebulized to 0, 6.25, 12.5, 25, 50, and 100 mg/ml of MeCh using the parameters tidal volume (10 ml/kg), frequency (150/min), and positive end-expiratory pressure (3 cm H_2_O).

### Immunostaining of airways for α-SMA

After sacrificing mice, lungs were inflated with 1 ml of 10% neutral buffer formalin using a tracheal catheter. The lungs were then dissected and placed in 10% neutral buffer formalin overnight. Following an additional day in 70% ethanol, the midsection of the left lung was then paraffin embedded. Each section was deparaffinized and rehydrated. Antigens were then unmasked using near boiling temperatures of 10 mM sodium citrate. Using 3% hydrogen peroxide, unmasked sections were quenched for endogenous peroxidase activity. After blocking with 5% goat serum in TBST, sections were immunostained with anti–α-SMA primary antibody (CST) at 1:400 dilution in 1% bovine serum albumin TBST overnight at 4 °C. Signal Stain DAB reagent (CST) was then used to stain the primary antibody. A species isotype control was used to identify any nonspecific staining. Slides were then counterstained with hematoxylin, dehydrated, and mounted. Using a 20× dry objective on Leica DMi8 microscope, slides were imaged. As previously described (64), α-SMA was quantified using ImageJ software by first outlining stained peribronchial area. This area was then normalized to the length of the basement membrane of the bronchiole. Slides were blinded during image acquisition, and each individual data point represents the average normalized α-SMA area from three different bronchioles from a single mouse.

### Peribronchial Masson’s trichrome staining of airways

Lung slices were deparaffinized and stained with Masson’s trichrome stain kit. Images were acquired using a 20× dry objective on Leica DMi8 microscope. Using ImageJ software, blue positive-stained areas around bronchioles were outlined and quantified. Slides were blinded during image acquisition, and each individual data point represents the average normalized blue Masson’s trichrome–stained area from three different bronchioles from a single mouse.

### Quantification of BAL cells and cytokines

Following methods previously described (83), the lungs of sacrificed mice were first inflated with 1 ml of PBS with 0.1 mM EDTA using a tracheal catheter. About 1 ml of BAL was then collected and centrifuged at 1500 rpm. The supernatants were stored for subsequent ELISA experiments at −80 °C. Ammonium–chloride–potassium buffer was then added to the cell pellet and incubated on ice for 5 min. After centrifuging the sample again, the cell pellet was resuspended with 500 μl of RPMI medium. Total BAL cell counts were recorded using a hemocytometer. For differential cell counts, 50,000 cells were loaded into a Cytofunnel (Thermo Fisher Scientific) and centrifuged for 10 min at 600 rpm. Slides were then stained using Diff-Quick stain kit. Differential cell counts were recorded based on morphology and staining patterns.

Using an ELISA kit (Thermo Fisher Scientific), mouse IgE was measured from supernatants of BAL following the manufacturer’s protocol. These measurements were normalized to total protein in these supernatants, which was measured using the Pierce Rapid Gold BCA Protein Assay.

### Statistical methods

All statistical analyses used GraphPad Prism 9 software (GraphPad Software, Inc). Data are represented as mean ± SEM. Experiments were repeated at least three independent times. For statistical analyses with only two experimental groups, two-sample *t* tests were performed, and for statistical analyses with more than two experimental groups, one-way ANOVA was performed with Dunnett’s method for multiple comparisons. For nonparametric distributions, normalcy was calculated using the Anderson–Darling test. For the nonparametric distributions, statistical significance was calculated using the Mann–Whitney test for analyses with only two experimental groups and the Kruskal–Wallis test for analyses with more than two experimental groups. ∗, ∗∗, and ∗∗∗ indicates *p* values of <0.05, <0.01, and <0.001, respectively. Differences were considered statistically significant when *p* < 0.05.

## Data availability

All raw data and raw unprocessed Western blots are provided in the supporting information. RNA-Seq data and metabolomics data are also provided in the supporting information.

## Supporting information

This article contains supporting information.

## Conflict of interest

The authors declare that they have no conflicts of interest with the contents of this article.

## Supporting information

metabolomics source data

R3_source data NCLX asthma post image analysis

Source data RNAseq
